# White Cells Facilitate Opposite- and Same-Sex Mating of Opaque Cells in *Candida albicans*

**DOI:** 10.1371/journal.pgen.1004737

**Published:** 2014-10-16

**Authors:** Li Tao, Chengjun Cao, Weihong Liang, Guobo Guan, Qiuyu Zhang, Clarissa J. Nobile, Guanghua Huang

**Affiliations:** 1 State Key Laboratory of Mycology, Institute of Microbiology, Chinese Academy of Sciences, Beijing, China; 2 University of Chinese Academy of Sciences, Beijing, China; 3 Department of Molecular and Cell Biology, School of Natural Sciences, University of California, Merced, Merced, California, United States of America; Institut Pasteur, France

## Abstract

Modes of sexual reproduction in eukaryotic organisms are extremely diverse. The human fungal pathogen *Candida albicans* undergoes a phenotypic switch from the white to the opaque phase in order to become mating-competent. In this study, we report that functionally- and morphologically-differentiated white and opaque cells show a coordinated behavior during mating. Although white cells are mating-incompetent, they can produce sexual pheromones when treated with pheromones of the opposite mating type or by physically interacting with opaque cells of the opposite mating type. In a co-culture system, pheromones released by white cells induce opaque cells to form mating projections, and facilitate both opposite- and same-sex mating of opaque cells. Deletion of genes encoding the pheromone precursor proteins and inactivation of the pheromone response signaling pathway (Ste2-MAPK-Cph1) impair the promoting role of white cells (*MTL* **a**) in the sexual mating of opaque cells. White and opaque cells communicate via a paracrine pheromone signaling system, creating an environment conducive to sexual mating. This coordination between the two different cell types may be a trade-off strategy between sexual and asexual lifestyles in *C. albicans*.

## Author Summary

In eukaryotic organisms, cells often undergo differentiation into distinct cell types in order to fulfill specialized roles. To achieve a certain function, different cell types may behave coordinately to complete a task that they may otherwise be incapable of completing independently. The human fungal pathogen *Candida albicans* can exist as two functionally and morphologically distinct cell types: white and opaque. The white cell type is thought to be the default state and may be the majority cell population in nature. However, only the minority opaque cells are mating-competent. In this study, we report that white and opaque cells show a coordinated behavior in the process of mating. When in the presence of opaque cells with an opposite mating type, white cells release sexual pheromones, and thus create an environment conducive for both opposite- and same-sex mating of opaque cells. The two cell types communicate via a paracrine pheromone signaling system. We propose that this communal coordination between white and opaque cells may not only support the fungus to be a successful commensal and pathogen in the host, but may also increase the fitness of the fungus during evolution over time.

## Introduction

Sexual reproduction is pervasive in eukaryotic organisms due to its propensity to permit genetic exchange, eliminate harmful mutations, and produce adaptive progeny to changing environments [1], [2]. It has been demonstrated to be critical for environmental adaptation, morphological transitioning, and virulence of human fungal pathogens [3], [4]. However, the evolutionary advantages of sexual over asexual reproduction in single-celled organisms are extremely complex when it comes to deconvoluting the interactions between host and pathogen [5]–[7]. For example, the three most frequently isolated human fungal pathogens – *Cryptococccus neoformans*, *Aspergillus fumigatus* and *Candida albicans* – have all maintained their mating machinery and are capable of undergoing sexual and/or parasexual reproduction, and yet their population structures appear to be largely clonal with little or no observable recombination [5]–[7]. It has been proposed that a balance between asexual and sexual reproduction may allow pathogenic fungi to generate clonal populations to thrive in their well-adapted environmental niches and to reproduce sexually and produce genetically diverse offspring in response to novel environmental pressures [6]. *C. albicans* has recently been shown to undergo opposite- and same-sex mating [8]–[10]. In this study, we demonstrate that morphological transitions play an important role in the control of sexual mating, and function to balance sexual and asexual lifestyles in *C. albicans*. This unique mode of sexual reproduction not only confers the fungus the ability to quickly adapt to the environment as a short-term strategy, but also provides a means to generate genetic diversity in response to unforeseen challenges during evolution over time.

There are three configurations of the mating type locus (*MTL* **a**/α, **a**/**a** and α/α) in *C. albicans*. The majority of natural isolates are **a**/α at the mating type locus [11]. *C. albicans* can frequently undergo a transition between two distinct cell types: white and opaque [12].To mate, *C. albicans* must first undergo a homozygosis at the mating type locus to become **a**/**a** and α/α, and then switch from the white to the opaque cell type [13]; only opaque cells can mate efficiently. Aside from mating-competency, white and opaque cells also differ in a number of other aspects, including global gene expression patterns, metabolic profiles, cellular appearances, and virulence properties in the host [12], [14]–. The white cell type is thought to be the default state since white cells are more stable than opaque cells at the host physiological temperature (37°C) and are also less vulnerable to stresses, antifungals and host immune system attacks [16]–[18].

Given that the white cell type is the default state and that the minority population of the opaque cell type is the only mating-competent form, one would hypothesize that mating in natural conditions would be rare. If this is the case, the many advantages of sexual reproduction over asexual reproduction in *C. albicans* would be very limited. This also raises an interesting question, that is, why does *C. albicans* undergo white-opaque switching, while still retaining such a costly sexual reproduction system? The discovery by Daniels et al. (2006) of the ability of opaque cells to signal white cells to form biofilms provides a clue to answer this question [19]. White and opaque cells may coordinate to regulate pathogenesis and resistance to environmental stresses through the development of biofilms. Recently, Park et al. (2013) reported that biofilms formed by white cells facilitate opaque cell chemotropism and thus increase mating efficiency in *C. albicans* [20]. In addition, pheromone has been shown to up-regulate the expression of a number of mating-associated genes in mating-incompetent white cells [19], [21]. In opaque cells (*MTL* **a**/**a**), α-pheromone induces the expression of both *MFA1* and *MFα*1 genes, which encode **a**- and α-pheromone precursors, respectively [21], [22]. Alby et al. (2009) further demonstrated that the addition of extracellular pheromone released by α opaque cells can induce same-sex mating in opaque **a** cells of *C. albicans* [10]. They found that the Bar1 protease plays a critical role in unisexual reproduction by controlling the autocrine pheromone signaling pathway [10]. Interestingly, species of the basidiomycete fungus *Cryptococcus* can also undergo opposite- and same-sex mating [23], [24]. Given that the population structures of these fungi are primarily clonal, unisexual reproduction may provide a long-term survival advantage, potentially increasing their ability to adapt to environmental changes.

Here we demonstrate that the interaction of white and opaque cells activates a paracrine pheromone signaling pathway in *C. albicans*. We further show that white cells facilitate both opposite- and same-sex mating of opaque cells. Given that the white phenotype is the default state and that opaque cells are less stable and more vulnerable in the host, our study provides additional clues to understanding how sexual mating in this organism is regulated. We suggest that the two cell types of *C. albicans* coordinate in order to balance the organism's commensal, pathogenic and sexual lifestyles.

## Results

### White “a” cells induce mating projection formation in opaque “α” cells

In several negative controls (e.g. the “WT, wh **a**×WT, op α” cross) performed in a mating assay, we observed that a very high proportion of opaque α cells formed mating projections, while the mating efficiency of the cross was extremely low (**Table S1**). One possible explanation for this low mating efficiency is that a small proportion of white **a** cells spontaneously switched to the opaque state to induce the formation of mating projections. To test this possibility, a mating assay of the cross of “*wor1*Δ/Δ, wh **a**×WT, op α” was performed and mating response was examined. The *wor1*Δ/Δ mutant is locked in the white phase because the white-opaque master regulator Wor1 is essential for opaque cell formation [25]–[27]. As shown in **Table S1**, similar to the cross of “WT, wh **a**×WT, op α”, the mating efficiency of the cross of “*wor1*Δ/Δ, wh **a**×WT, op α” was also very low, while the proportion of opaque α cells with mating projections was over 75%. These results indicate that white **a** cells induce opaque α cells to form mating projections in *C. albicans*, but do not increase the mating efficiency of the cross between white cells and opaque cells.

**Figure 1:**
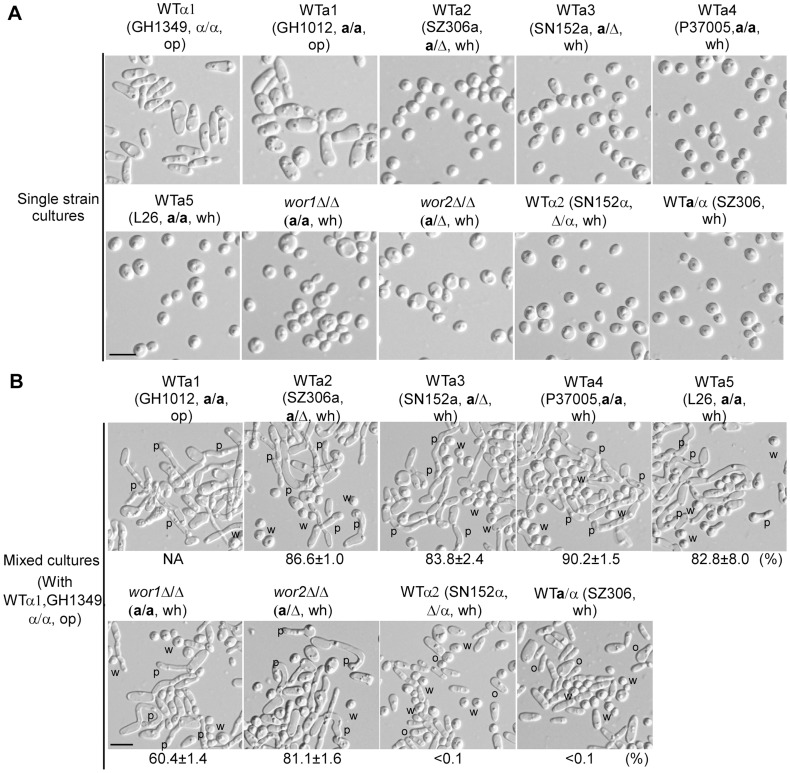
White a cells induce mating projection formation in opaque α cells in mixed cultures of white (a) and opaque (α) cells. Strains: *wor1*Δ/Δ (GH1248, *MTL*a/a); *wor2*Δ/Δ (MMY627, *MTL*a/Δ). (A) Cellular images of single strain cultures. WTα1, GH1349, *MTL*α/α; WTα2, SN152α, *MTL*Δ/α. Five WT **a** strains of different background (WTa1 to WTa5) and the *wor1*Δ/Δ and *wor2*Δ/Δ mutants were used. SZ306, *MTL* **a**/α, served as a control. (B) Cellular images of mixed cultures. 4×10^6^ opaque α cells (GH1349) were mixed with 4×10^6^ cells of different background as indicated. The mixtures were spotted onto Lee's glucose medium and cultured at 25°C in air for 24 hours. Cellular images and percentages of opaque α cells (GH1349) with mating projections are shown. Cells with at least one mating projection were counted. The mixture of opaque α cells (GH1349) and opaque **a** cells (GH1012) served as a positive control. The percentage of opaque α cells (GH1349) with mating projections in the opaque **a**×α mixture is not shown since both α and **a** cells formed mating projections. SZ306 (**a**/α) and SN152α served as negative controls. NA, not available. W (or wh), white; O (or op), opaque; P, mating projection. Scale bar, 10 µm.

To further confirm this phenomenon, we tested the effect of white **a** cells of four strains with different genetic backgrounds on the induction of mating projection formation of opaque α cells. The assay was performed on nutrient solid agar (Lee's glucose medium). As shown in **Figure 1**, over 75% of opaque α cells formed mating projections in all of the mixed cultures containing white **a** cells of the wild type strains. Consistently, cells of the *wor1*Δ/Δ and *wor2*Δ/Δ mutants, which are “locked” in the white phase under this culture condition, also induced mating projection formation in opaque α cells (**Figure 1B**). Opaque **a** cells served as a positive control, and white **a**/α cells and white α cells served as negative controls. As expected, opaque **a** cells induced mating projection formation in opaque α cells, while white **a**/α cells and white α cells did not. The images of single strain cultures and the ratios of opaque cells with mating projections are shown in **Figure 1A and 1B**, respectively. Consistently, white cells of another clinically independent WT **a** strain (SZ306a, **a**/Δ) and the *wor1*Δ/Δ mutant (GH1248, **a**/**a**) also induced mating projection formation in opaque α cells when cultured in liquid medium (**Figure S1**). These results indicate that the induction of mating projections of opaque cells by white cells is a general feature of clinical isolates of *C. albicans*.

We next examined the effect of the ratio of opaque α cells to white **a** cells in the mixed cultures on the formation of projections in opaque α cells. White **a** cells of the WT (SZ306a), *wor1*Δ/Δ and *wor2*Δ/Δ mutants were tested. As shown in **Figure S2**, the percentages of projections in opaque α cells were inversely related to the ratio of initial cell numbers of opaque α cells to white **a** cells added to the mixture. To ensure that the observed projections of the opaque cells were indeed mating projections, 4′-6-diamidino-2-phenylindole (DAPI)-DNA staining assays were performed. A single nucleus was observed by fluorescence microscopy in cells with newly formed projections (**Figure S3**). These results provide additional evidence that white **a** cells can induce mating projection formation in opaque α cells.

### Opaque “α” cells induce *MFA1* expression in white “a” cells

**Figure 2:**
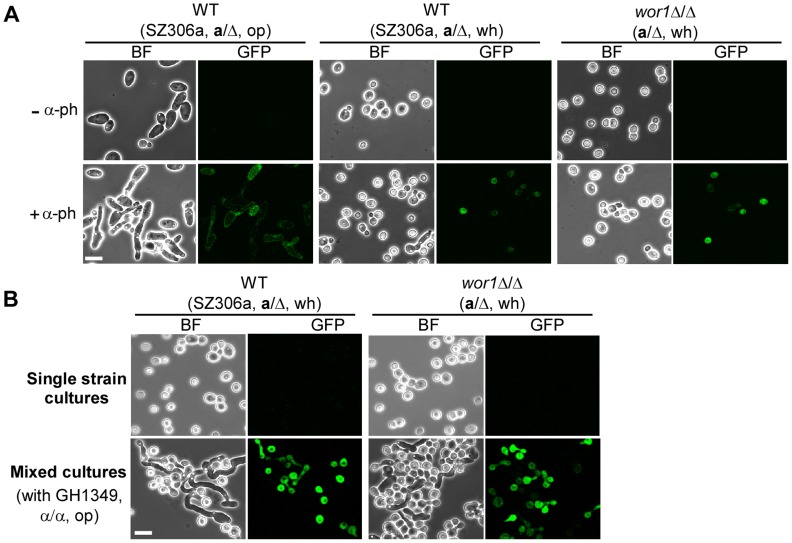
*MFA1* expression in white a cells. Two reporter strains GH1600 (SZ306MFA1p-GFP) and GH1603 (*wor1*Δ/ΔMFA1p-GFP), in which *GFP* gene was under the control of the *MFA1* promoter, were used. wh, white; op, opaque. Strains used: GH1600 and GH1603. (A) α-Pheromone induces *MFA1* expression in white **a** cells. Cells were first grown at 25°C for 36 hours to stationary phase and inoculated into fresh Lee's glucose medium (1×10^7^ cells/ml). α-Pheromone peptide was added every two hours to the cultures over an eight-hour period. The final concentration of α-pheromone peptide was 8×10^−6^ M. Expression of GFP proteins was examined with a fluorescence microscope. Opaque **a** cells of strain SZ306MFA1p-GFP served as a control. Images of untreated cells are shown in top panels. “−α-ph”, no α-pheromone added (upper panel); “+α-ph”, α-pheromone added (lower panel). BF, bright field. Scale bar, 10 µm. (B) Opaque α cells induce *MFA1* expression in white **a** cells. 4×10^6^ white **a** cells of each tester strain indicated were mixed with equal number of opaque α cells (GH1349 *MTL*α/α). The mixtures were spotted onto Lee's glucose medium and incubated at 25°C for 24 h. Expression of GFP proteins in white cells of the tester strains was examined with a fluorescence microscope. Images of single strain cultures (white cells of the tester strains) are shown in the upper panel. Mixed cultures are shown in the lower panel. BF, bright field. Scale bar, 10 µm.

*MFα1* is constitutively expressed in opaque α cells examined over a 48-hour growth period (**Figure S4**) [28]–[30], while *MFA1* is poorly expressed in opaque **a** cells [22]. When treated with α-pheromone, *MFA1* is induced in opaque **a** cells [22]. We hypothesized that *MFA1* may also be induced in white **a** cells upon addition of α-pheromone to the medium or through production by opaque α cells. If true, in a mixed culture of white **a** cells and opaque α cells, the α cells should form mating projections as a result of exposure to **a**-pheromone produced by white **a** cells. To test this hypothesis, two reporter strains (SZ306MFA1p-GFP and *wor1*Δ/ΔMFA1p-GFP), in which a GFP coding sequence was integrated at the *MFA1* locus and controlled by the *MFA1* promoter, were constructed. As shown in **Figure 2A**, α-pheromone clearly induced *MFA1* expression in a proportion of white cells of the two reporter strains as indicated by the GFP fluorescence. Opaque cells of the SZ306a-MFA1p-GFP strain treated with α-pheromone served as a positive control. In the mixed cultures of white **a** cells and opaque α cells (**Figure 2B**), the expression of GFP in the two reporter strains was also clear. However, the GFP fluorescence was not observed in the single strain cultures. These results indicate that the presence of α-pheromone, either from its addition to the medium or produced by opaque α cells, is able to induce the expression of *MFA1* in white **a** cells.

### *MF*α*1* expression in white “α” cells

*MF*α*1* is constitutively expressed in opaque α cells, but not in white α cells (**Figure S4 and S5**). We next tested whether the expression of *MF*α*1* could be induced in white α cells by **a**-pheromone. A MFαp-GFP reporter strain was constructed as described in the *Materials and Methods* section. Considering that the expression level of *MFA1* is extremely low in opaque **a** cells in the absence of α-pheromone, opaque α cells were added to one of the mixed cultures to provide α-pheromone to the mixture. As shown in **Figure S5**, the expression of *MF*α*1* in white α cells was induced as indicated by the GFP fluorescence in the mixed cultures of both “white α cells + opaque **a** cells” and “white α cells + opaque **a** cells + opaque α cells”. We were surprised that *MF*α*1* was induced in the former culture since the expression level of *MFA1* in opaque **a** cells is extremely low in the absence of opaque α cells. There are two possible explanations for this finding. First, low levels of **a**-pheromone secreted by opaque **a** cells may have induced *MF*α*1* expression in white α cells. Alternatively, a small proportion of white α cells may have spontaneously converted to the opaque form and induced the expression of *MFA1* in opaque **a** cells, which in turn induced *MF*α*1* expression in white α cells. Overall, these results suggest that white α cells can produce pheromone and may play a similar role as white **a** cells in promoting an environment conducive to mating.

### Pheromones are required for communication between white and opaque cells

**Figure 3:**
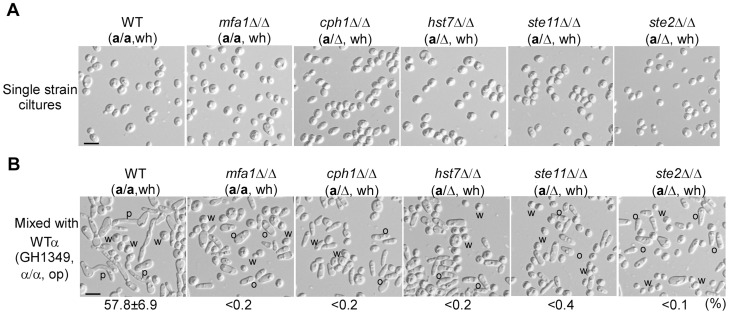
a-Pheromone (MFa1) and the α-pheromone response pathway (Ste2-MAPK-Cph1) of a cells are required for a white cells to induce mating projection formation in α opaque cells (GH1349). Strains used: GH1013, GH1609, GH1610, GH1613, GH1614, and GH1615. (A) Cellular images of single strain cultures. (B) Cellular images of mixed cultures. Opaque α cells (GH1349) were mixed with white **a** cells of different mutants as indicated. This experiment was performed as described in Figure 1B. Percentages of opaque α cells (GH1349) with mating projections are shown below the images. W, white; O, opaque; P, mating projection. Scale bar, 10 µm.

We hypothesized that α-pheromone produced by opaque α cells could induce *MFA1* expression in white **a** cells. We, therefore, deleted the *MF*α*1* gene, encoding the precursor protein of α-pheromone, in a WT α strain (α/α, GH1617). We found that opaque cells of the *mf*α*1*Δ/Δ mutant could not induce *MFA1* expression in white **a** cells (**Figure S6**). Consistently, white **a** cells were unable to induce mating projection formation in opaque cells of the *mf*α*1*Δ/Δ mutant (**Figure S6**). To test whether **a**-pheromone is essential for the communication between white and opaque cells, we next deleted *MFA1* in a WT **a** strain (**a**/**a**, GH1609). As shown in **Figure 3**, white cells of the *mfa1*Δ/Δ mutant failed to induce mating projection formation in opaque α cells. These results indicate that pheromones act as signaling molecules in the interaction between white and opaque cells.

### The Ste2-MAPK-Cph1 pheromone response pathway in white “a” cells is required for the induction of mating projection formation of opaque “α” cells

The pheromone receptors (Ste2 and Ste3) and the downstream MAPK pathway are highly conserved in the regulation of sexual mating in fungi [5], [31], [32]. In *Saccharomyces cerevisiae*, *Cryptococcus neoformans*, and *C. albicans*, the MAPK pathway governs both mating and filamentation [33]–[36]. The downstream transcription factor Cph1 in *C. albicans*, a homolog of Ste12 in *Saccharomyces cerevisiae*, is also essential for mating [37], [38]. The Ste2/3-MAPK-Cph1 pathway is involved in pheromone response in both white and opaque cells of *C. albicans* [39], [40]. Given this information, we examined the role of this pathway in the interactions between white and opaque cells. As shown in **Figure 3**, deletion of genes (*STE2*, *STE11*, *HST7* and *CPH1*) of the α-pheromone response pathway in white **a** cells blocked the induction of mating projection formation by opaque α cells. The morphological images of single strain cultures and the ratios of projected opaque α cells are presented in **Figure 3A and 3B**, respectively. These results suggest that both the α-pheromone response pathway and **a**-pheromone are required for white **a** cells to induce mating projection formation in opaque α cells.

### The Ste3-MAPK-Cph1 pheromone response pathway in opaque “α” cells is required for white “a” cells to induce mating projections in opaque cells

**Figure 4:**
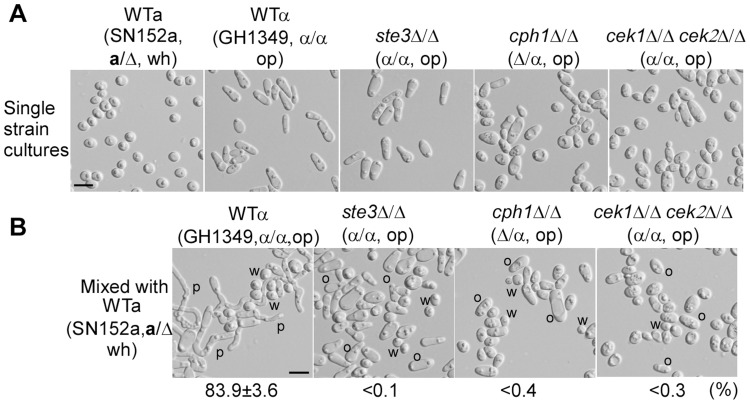
The a-pheromone response pathway (Ste3-MAPK-Cph1) of α opaque cells are required for a white cells (WTa, SN152a/Δ) to induce mating projection formation in α opaque cells. Strains used: SN152a, GH1349, GH1611, GH1616, and GH1247. (A) Cellular images of single strain cultures. (B) Cellular images of mixed cultures. White cells of the WTa strain were mixed with opaque α cells of different mutants as indicated. The assay of mating projection formation was performed as described in Figure 1B. Percentages of opaque α cells with mating projections are shown below the images. The mixed culture of opaque α cells (GH1349) and white **a** cells (WTa) served as a positive control. W, white; O, opaque; P, mating projection. Scale bar, 10 µm.

The Ste3-MAPK-Cph1 pathway is required for mating of opaque cells [22], [38], [41] and is essential for pheromone-induced biofilm formation [39]. We next tested the ability of the *ste3*Δ/Δ, *cph1*Δ/Δ and *cek1*Δ/Δ *cek2*Δ/Δ opaque α cell mutants to form mating projections when co-cultured with white **a** cells. As shown in **Figure 4**, opaque cells of these three mutants failed to form mating projections, while over 80% of opaque cells of the WT control formed mating projections.

### White cells facilitate opposite-sex mating of opaque cells

Sexual pheromones are essential for mating in *C. albicans*. We next tested whether white **a** cells could facilitate mating of opaque cells by producing **a**-pheromone, causing an increase in pheromone concentration to the level required to activate the mating signaling process. We designed an opposite-sex mating system, which contained 1×10^4^ opaque **a** cells, 3.2×10^6^ opaque α cells and 4.8×10^6^ “helper” white cells. The system was so designed for the following reason. Mating between opaque **a** and α cells in the presence of high cell densities may increase mating efficiency. This system should amplify the promoting function of white cells by using less opaque **a** cells in order to reduce the high mating efficiency between opaque cells. White cells of the **a**/α strain (BWP17), *wor1*Δ/Δ (**a/a**), *mf*a*1*Δ/Δ (**a/a**), and *wor1*Δ/Δ *mfa1*Δ/Δ (**a/a**) mutants served as the “helper” white cells in the mating system. The *wor1*Δ/Δ mutant was used for this experiment because cells of this strain are “locked” in the white phase under all conditions tested [25]–[27]. As shown in **Table 1**, the mating efficiency of the cross when the *wor1*Δ/Δ (**a**/**a**) mutant served as the “helper” was about six-fold higher than that of the other three crosses with the **a**/α strain, *mfa1*Δ/Δ, and *wor1*Δ/Δ *mfa1*Δ/Δ mutants as the “helpers”. To further verify these results, we tested the roles of white cells of the *wor1*Δ/Δ mutants in facilitating opposite-sex mating of opaque cells in two additional genetic backgrounds (derivatives of SZ306 and SN152). Consistently, compared with the **a**/α strains (the WT and *wor1*Δ/Δ mutant), white **a** cells of the *wor1*Δ/Δ mutant (**a**/Δ) increased mating efficiencies by six- to nine-fold (**Table 1**). Consistently, deletion of *MFA1* in white cells blocked this facilitation role in opposite-sex mating of opaque cells (**Table 1**, *mfa1*Δ/Δ and *wor1*Δ/Δ *mfa1*Δ/Δ mutants).

**Table 1:** White “a” cells facilitate opposite-sex mating of opaque cells.

“Helper” white cells	Mating efficiency of a-op(his1*Δ/Δ*)×α-op(arg4*Δ/Δ*)
BWP17 (**a**/α, *ura3Δ/Δ*)	(3.6±0.1)×10^−3^
GH1013(**a**/**a**, *ura3Δ/Δ mfa1Δ/Δ*)	(4.0±1.5)×10^−3^
GH1248(**a**/**a**, *ura3Δ/Δ wor1Δ/Δ mfa1Δ/Δ*)	(3.2±1.6)×10^−3^
GH1248(**a**/**a**, *ura3Δ/Δ wor1Δ/Δ*)	(1.8±1.0)×10^−2^ **(∼6-fold increase)**
SZ306 (**a**/α, *ura3Δ/Δ*)	(5.2±1.6)×10^−3^
SZ306 (**a**/α, *ura3Δ/Δ wor1Δ/Δ*)	(3.8±0.5)×10^−3^
SZ306 (**a**/*Δ, ura3Δ/Δ wor1Δ/Δ*)	(2.1±0.8)×10^−2^ **(∼6-fold increase)**
SN152 (**a**/α, *ura3Δ/Δ arg4Δ/Δ his1Δ/Δ leu2Δ/Δ*)	(4.6±1.7)×10^−3^
SN152 (**a**/α, *ura3Δ/Δ arg4Δ/Δ wor1Δ/Δ*)	(3.9±1.3)×10^−3^
SN152a (**a**/Δ, *ura3Δ/Δ arg4Δ/Δ wor1Δ/Δ*)	(3.4±1.4)×10^−2^ **(∼9-fold increase)**
No “helper” white cells	(3.8±1.0)×10^−3^

Briefly, 4.8×10^6^ of “helper” white cells of different genetic backgrounds (as indicated) were added to the mating mixture (1×10^4^ opaque **a** cells plus 3.2×10^6^ opaque α cells). The mating mixtures were spotted onto Lee's glucose medium plates and cultured at 25°C for 48 hours. The mating mixtures were replated onto SD-histidine-uridine, SD-arginine-uridine and SD-uridine-arginine-histidine media for prototrophic selection. All the “helper” strains are *ura3*Δ/Δ mutants and are unable to grow on media without uridine. Mating tester strains: **a**-op (as GH1013, but *his1*Δ/Δ *URA3+ARG4+*) and α-op (GH1349, arg4*Δ/Δ*). Three groups of white “helper” strains of different genetic backgrounds (including BWP17, SZ306, SN152 and their derivatives) were used.

To further demonstrate that white **a** cells are able to facilitate opposite-sex mating of opaque cells by producing **a**-pheromone, we designed another mating system. We deleted the *MFA1* gene in opaque cells of the “*MTL* **a**” mating partner (*mfa1*Δ/Δ, **a**/**a**), resulting in the failure to produce **a**-pheromone. White cells of the **a**/α strains (the WT and *wor1*Δ/Δ mutant) and the **a** strain (*wor1*Δ/Δ, **a**/Δ) served as the “helpers” in the mating cross of the “**a**-op (*mfa1*Δ/Δ, **a**/**a**)×α-op (WT, GH1349, α/α)”. Strains of two different genetic backgrounds (SZ306 and SN152) were also used as “helpers”. As shown in **Table 2**, mating of the “**a**-op (*mfa1*Δ/Δ, **a**/**a**)×α-op (WT, GH1349, α/α)” cross only occurred in the presence of white **a** cells. These results indicate that white **a** cells facilitate opposite-sex mating of opaque cells by producing **a**-pheromone.

**Table 2:** White “a” cells promote same-sex mating of opaque cells (α×α) and opposite-sex mating between “a” opaque cells of the *mfa1*Δ/Δ mutant and “α” opaque cells (a-*mfa1*Δ/Δ×α).

“Helper” white cells	Mating efficiency	
	α-op × α-op	a-op(*mfa1Δ/Δ*)×α-op
SZ306 (**a**/α, *ura3Δ/Δ*)	<5×10^−9^	<9×10^−8^
SZ306 (**a**/α, *ura3Δ/Δ wor1Δ/Δ*)	<4×10^−9^	<6×10^−8^
SZ306 (**a**/*Δ, ura3Δ/Δ wor1Δ/Δ*)	(2.7±0.5)×10^−7^	(1.4±1.1)×10^−6^
SN152 (**a**/α, *ura3Δ/Δ arg4Δ/Δ his1Δ/Δ leu2Δ/Δ*)	<4×10^−9^	<6×10^−9^
SN152 (**a**/α, *ura3Δ/Δ arg4Δ/Δ wor1Δ/Δ*)	<5×10^−9^	<7×10^−8^
SN152a (**a**/*Δ*, *ura3Δ/Δ arg4Δ/Δ his1Δ/Δ leu2Δ/Δ*)	(1.8±0.1)×10^−6^	(1.6±0.6)×10^−6^
SN152a (**a**/Δ, *ura3Δ/Δ arg4Δ/Δ wor1Δ/Δ*)	(1.1±0.4)×10^−6^	(2.5±1.0)×10^−6^
No “helper” white cells	<5.1×10^−10^	<1.2×10^−9^

The “α-op×α-op” cross: 9.6×10^7^ white cells of “helper” strains (60%) were mixed with 3.2×10^7^ opaque cells of SZ306α (*Δ*/α, *ura3Δ/Δ*, 20%) and 3.2×10^7^ opaque cells of GH1349 (α/α, *arg4Δ/Δ*, 20%). The “**a**-op(*mfa1Δ/Δ*)×α-op” cross: 9.6×10^7^ white cells of “helper” strains (60%) were mixed with 3.2×10^7^ opaque **a** cells of GH1609 (**a**/**a**, *mfa1Δ/Δ, ura3Δ/Δ* 20%) and 3.2×10^7^ opaque cells of GH1349 (α/α, *arg4Δ/Δ*, 20%). The mating mixtures were spotted onto Lee's glucose medium plates and cultured at 25°C for 5 days. Then, mixed cells were replated onto SD medium for prototrophic selection. SZ306 (**a**/Δ, *ura3Δ/Δ wor1Δ/Δ*) is locked in the white phase since it is a *wor1Δ/Δ* mutant. In the mating mixture where SZ306a served as the “helper” strain, white cells of SZ306a were able to mate with opaque α cells at extremely low efficiencies. The progeny of this mating cross were able to grow on selectable plates because SZ306a only carries a *ura3*-auxotropic marker. To exclude these mating products from our calculations, all mating progeny colonies were subject to PCR verification, and only correctly validated colonies were used in the calculation. “<”, indicates that “mating efficiency” is less than a certain number or no progeny colonies were observed on selectable plates.

### White cells facilitate same-sex mating of opaque cells

Opaque cells of *C. albicans* can undergo same-sex mating in the presence of the opposite mating pheromone [10]. We, therefore, predicted that white **a** cells could facilitate same-sex mating of opaque α cells by producing **a**-pheromone in a co-cultured mating system. As shown in **Table 2**, opaque α cells of SZ306α and GH1349 (α/α) were unable to mate when white cells of the WT**a**/α (mating efficiency <5×10^−9^) or *wor1*Δ/Δ (**a**/α) mutant (mating efficiency <4×10^−9^) served as the “helper” strains. However, the mating efficiency was increased to (2.7±0.5)×10^−7^ (over 54 fold) when white **a** cells of the *wor1*Δ/Δ mutant (**a**/Δ, a derivative of SZ306) served as the “helper” strain. To further validate these results in another genetic background, we performed the same mating assays using white cells of the SN152 background strain as the “helpers”. Consistently, compared with the controls (when **a**/α cells severed as “helpers”), the mating efficiency was dramatically increased when white **a** cells of SN152a (over 450-fold increase) or the *wor1*Δ/Δ mutant (**a**/Δ, a derivative of SN152, over 220-fold increase) served as the “helpers” (**Table 2**). Consistently, in the absence of white α cells, same-sex mating was unable to occur in opaque **a** cells (**Figure S5**), suggesting that white α cells are also capable of promoting same-sex mating of opaque **a** cells.

### White cells facilitate sexual mating of opaque cells in a mouse skin infection model

**Figure 5:**
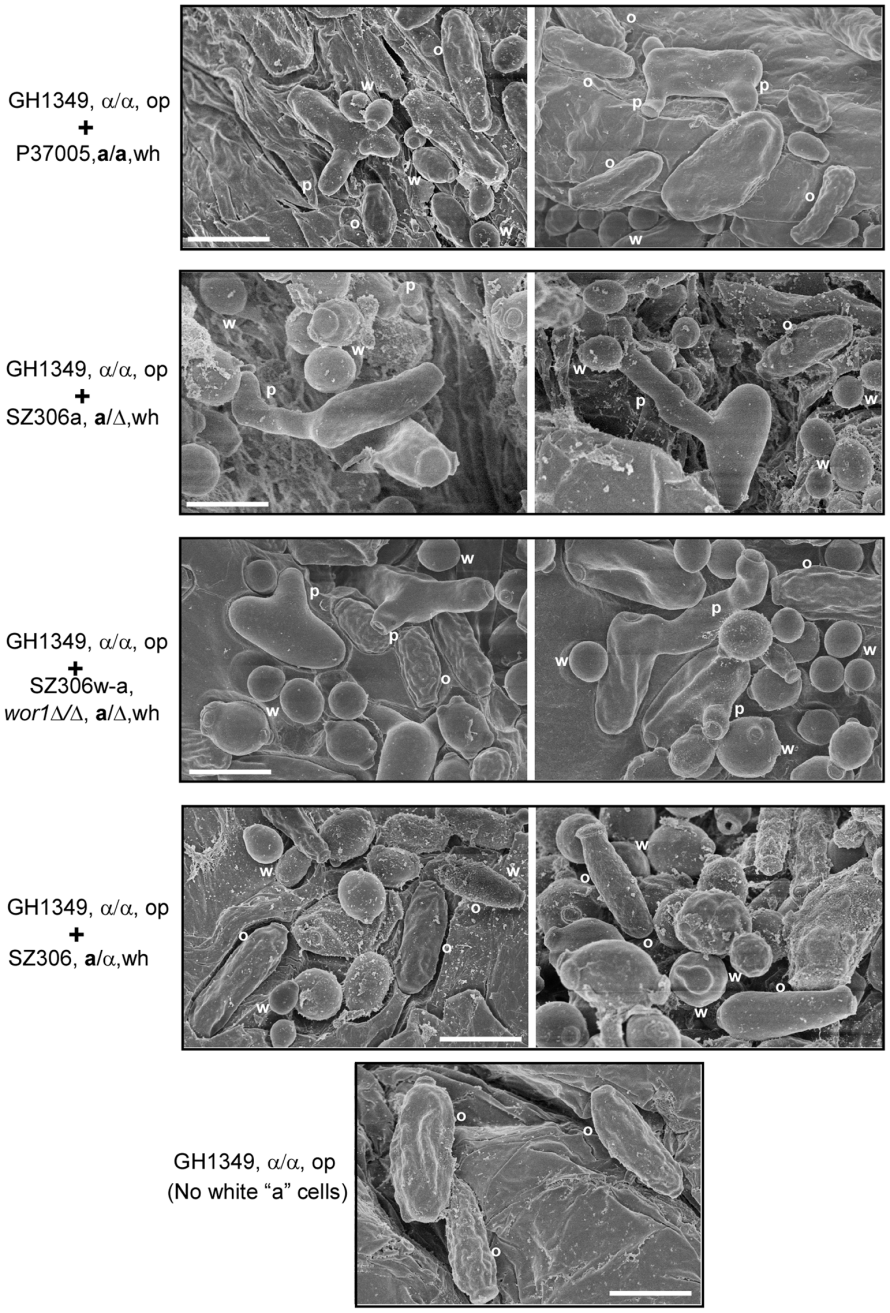
White a cells induce mating projection formation in opaque α cells in a mouse skin infection model. 1×10^7^ opaque α cells (GH1349) and 1×10^7^ white “helper” cells were mixed and spotted onto the skin of newborn mice for infection. The *wor1*Δ/Δ mutant (SZ306w-a), WT “**a**” (P37005 and SZ306a) and “**a**/α” strains (SZ306) were used for induction of mating projections. Opaque α cells (GH1349) alone (no white “helper” control) was used as a control. Representative SEM images are shown. W, white cells; O, opaque cells; P, mating projections. Scale bar, 10 µm.

To evaluate the in vivo relevance of our findings, we next tested whether white cells could facilitate opaque cell mating in a mammalian host. As shown in **Figure 5**, white **a** cells of the WT strains or the *wor1Δ/Δ* mutant strain induced the development of mating projections in opaque α cells. However, in the absence of white **a** cells or in the presence of white **a**/α cells, opaque α cells were unable to develop mating projections on the mouse skin. Quantitative mating assays also demonstrated that white **a** cells promoted opaque cell mating in this mouse skin infection model (**Table S2**). These results demonstrate that white cells are capable of facilitating opaque cell mating in a natural environmental niche.

### Global analysis of pheromone-regulated gene expression in white cells

Although white cells can be induced to produce pheromone, they are unable to mate (**Table S1**), suggesting that the pheromone response pathway of white cells is different from that of opaque cells. To explore how white cells respond to pheromone and how white cells create an environment conducive for opaque cell mating, we performed RNA-Seq analysis to investigate the global gene expression profile in white **a** cells in response to α-pheromone. Although gene expression profiling of white cells in response to pheromone have been previously published [21], [28], [42], these studies used white cells of wild type strains, which are opaque-competent (able to spontaneously switch from the white to the opaque phase). Because these strains were switching competent, a small proportion of opaque cells in the population could confound the results. In order to mitigate the effects of switching on the gene expression profile in response to pheromone treatments, we used the *wor1Δ/Δ* mutant (GH1602), which is “locked” in the white phase [25]–[27], for our RNA-seq experiment We believe that this dataset improves and strengthens the already published datasets, and more accurately reflects the effects of pheromone treatment on white cells exclusively. As shown in **Table 3**, 75 genes involved in a number of biological aspects were up-regulated and 124 genes were down-regulated in the presence of α-pheromone (using a two-fold cutoff). Our key findings are summarized below: (i) We observed differential expression in a subset of the mating-related or pheromone receptor-MAPK signaling pathway genes, including *MFA1*, *HST6*, *FAV1*, and *STE2*. Consistent with our quantitative real-time PCR (Q-RT-PCR) and MFA1p-GFP reporter results (**Figure 2** **and S7**), the expression of the *MFA1* gene was up-regulated hundreds of fold when treated with α-pheromone. Q-RT-PCR assays were performed to verify the expression levels of *MFA1*, *STE2*, and *STE3*, in white cells of the WT **a**/α, WT **a**, and *wor1Δ/Δ* mutant strains, as well as opaque **a** cells (**Figure S7 and S8**). (ii) A number of mating-related genes up-regulated by pheromone in opaque cells were not up-regulated in white cells [21], [28], [42]. These genes include *FIG1, FUS1, CEK1, CEK2, FAR1, CPH1*, and *HST6*. This result suggests that the mating and cell fusion pathways are not fully activated by pheromone in white cells as is the case in opaque cells, and provides an explanation as to why white cells are unable to mate. (iii) We observed a reduction in metabolism-related genes in the presence of α-pheromone, especially for nucleotide, lipid and fatty acid metabolism as well as for genes encoding ribosomal proteins and transporters. (iv) We also found that genes encoding cell surface proteins, which are involved in cohesion, adhesion, and biofilm formation, were differentially regulated by α-pheromone. We validated the expression levels of eight genes using quantitative RT-PCR assays (**Figure S8C**). Some pheromone-regulated genes observed in our study were also identified in previous studies performed in different strain backgrounds [19], [21], [42]. A detailed functional categorization and description of the differentially expressed genes in response to pheromone are presented in **Figure S8** and **Table S3**.

**Table 3:** Pheromone-regulated genes in white cells.

Category	Up-regulated genes	Down-regulated genes
**α-pheromone response proteins**	(**6**): *ASG7, MFA1, HST6, FAV1, RAM2, STE2*	(**3**): *MCD1, DAG7, PCL1*
**Carbohydrate metabolism**	(**13**): *IFE2, HGT6, XYL2, HXK2, OSM1, SCS7, ADH1, ADH5, HEM13, GAL10, FBA1, DLD2, MNN1*	(**33**): *PXP2, NCE103, HGT10, HGT12, HGT16, HGT17, CTN1, ACO1, RHD1, FAA2-3, ALD5, ALD6, ICL1, FAA21, CAT2, SDH12, SPS20, POX1-3, FUM12, CIT1, ADH2, ALK8, MLS1, HXT5, PMT2, PDC12, MAL2, GCV1, GFA1, LYS22, TES15, IDP2, ACH1*
**Lipid and fatty acid metabolism**	(**2**): *RTA4, ERG251*	(**8**): *FOX2, FOX3, FAD3, ERG6, ALG11, PEX11, DPP1, FAD2*
**Nitrogen, sulfur and amino acid metabolism**	(**14**): *SUL2, MET1, MET2, MET3, MET10, MET14, CRP1, YHB1, SSU1, ECM17, GAC1, MUP1, GCS1, GAP1*	(**7**): *GAP2, GDH3, GLT1, HIS3, GCV2, LYS5, CAN1*
**Nucleotide metabolism**	(**1**): *RNR22*	(**5**): *CDG1, RNH35, OGG1, RNR1, GUK1*
**Cohesion, adhesion, and biofilm formation**	(**1**): *PBR1*	(**3**): *ALS2, ALS4, SIM1*
**Protein activity regulation**	(**1**): *NAT4*	(**0**)
**Cell fate (cell growth, differentiation, cell surface)**	(**11**): *ECE1, HWP1, IHD1, PGA4, PGA7, PGA10, PGA18, PGA48, PGA58, UEC1, RBT5*	(**15**): *CCN1, PGA6, GIN4, MCD4, ECM331, RBR1, PMI1, CHT2, CDC10, CDC11, CDC47, PRA1, CLA4, ROT1, OSH3*
**SAPs & LIPs**	(**1**): *SAP30*	(**1**): *LIP8*
**Energy**	(**5**): *EBP1, PRX1, OYE23, BNA4, ARH2*	(**0**)
**Mating type & cell cycle**	(**1**): *CDC6*	(**8**): *MCM2, MCM3, MCM6, CDC46, CDC54, SWI6, POL1, SPC34*
**Stress response/Drug resistance**	(**4**): *HSP30, HSP31, RBT4, CYS3*	(**2**): *FMO1, CAT1*
**Ionic homeostasis**	(**3**): *FET34, COX17, CIP1*	(**4**): *FTR2, CFL4, CFL5, SEF2*
**Transcription**	(**7**): *HMS1, UME7, BRG1, DEF1, CPH2, RAS2, ZCF3*	(**4**): *TRY4, TRY5, CRZ1, PPR1*
**Transporters**	(**5**): *QDR1, OPT7, PTR2, ALP1, ITR1*	(**15**): *JEN1, JEN2, NAG3, NAG4, ANT1, SFC1, FRP3, VRG4, CDR1, SNQ2, ZRT1, PXA2, CDR2, MDR1, ARG11*
**Ubiquitination**	(**0**)	(**1**) *PEX4*
**Ribosome**	(**0**)	(**15**): *RPL3, RPL5, RPL8B, RPL9B, RPL11, RPL15A, RPL82, RPS3, RPS7A, RPS12, RPS19A, RPS20, RPS21, RPP1A, ASC1*

Strain used: the *wor1*Δ/Δ mutant (**a**/Δ, GH1602). Total RNA was extracted from α-pheromone-untreated or treated cells and used for RNA-Seq analysis. Genes are grouped by functional category according to [15], [50]. Differentially expressed genes using a two-fold relative expression cutoff are shown. Numbers of up- or down-regulated genes are indicated in the brackets.

## Discussion

White and opaque cells of *C. albicans* are two distinct cell types differing in a number of biological aspects [14], [16], [18]. Given that only opaque cells are mating-competent [13] and that white cells are the majority population in nature [18], the relationship between white-opaque transitions and sexual mating in *C. albicans* is extremely complex. These facts also raise several intriguing questions. Why is the white-opaque switch required for mating in *C. albicans*? What roles do white cells play in the process of sexual reproduction? How do white and opaque cells communicate?

The discovery of pheromone-induced biofilm formation in white cells of *C. albicans* [19], provides some intriguing clues to address these questions. It was suggested that biofilm formation by *MTL*-homozygous white cells in turn facilitate opaque cell mating [19], [20]. The white cell biofilm (or “sexual biofilm”) formed by *MTL*-homozygous white cells is distinct from that formed by *MTL*-heterozygous (**a**/α) cells. For example, the former was reported to be more permeable than the latter and to form gradients of pheromone for chemotropism [40].

**Figure 6:**
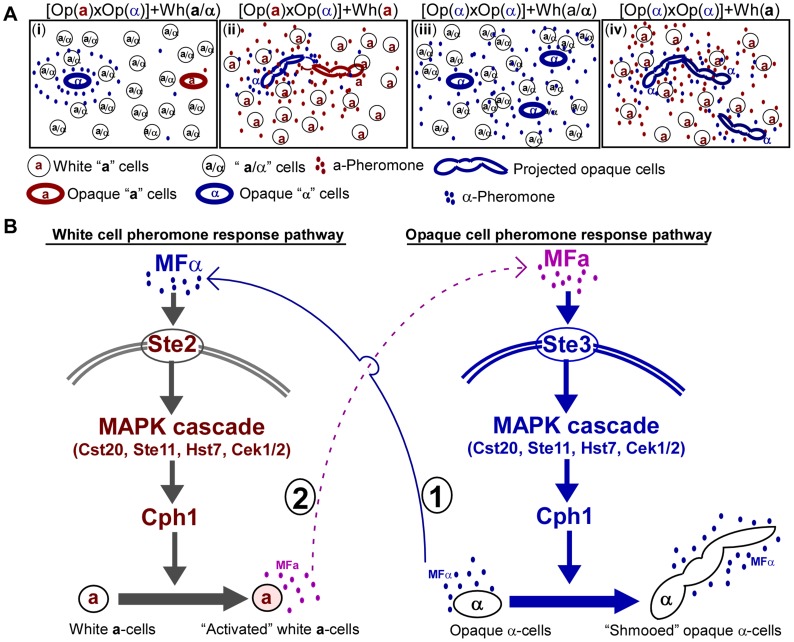
Schematic models for the role of white cells in opposite- and same-sex mating of opaque cells. (A) The majority of white cells (**a**) secrete pheromone to facilitate opposite- and same-sex mating of the minority opaque cells. (i) When the majority are **a**/α cells, sexual mating between the minority of opaque **a** and α cells is rare. Opaque α cells secrete α-pheromone constitutively, while opaque **a** cells do not secrete **a**-pheromone without exposure to a certain level of α-pheromone. When both opaque **a** and α cells are rare, the threshold for pheromone and mating responses is typically not reached. (ii) When the majority are white **a** cells, sexual mating between the minority opaque **a** and α cells can occur. Opaque α cells secrete α-pheromone and induce the majority of white **a** cells to secrete **a**-pheromone. Thus, **a**-pheromone in turn induces opaque α cells to form long mating projections, which facilitate them to reach opaque **a** mating partners. (iii) White **a**/α cells do not induce mating projections and do not facilitate same-sex mating of opaque α cells. (iv) White **a** cells facilitate same-sex mating of opaque α cells. Opaque α cells secrete α-pheromone and induce white **a** cells to secrete **a**-pheromone. In turn, **a**-pheromone induces opaque α cells to form mating projections and undergo same-sex mating. (B) Schematic model depicting the crosstalk between white and opaque pheromone response pathways. First, opaque α cells constitutively secrete α-pheromone and activate the white pheromone response pathway to induce **a**-pheromone secretion in **a** cells. Then, **a**-pheromone secreted by white **a** cells activates the opaque pheromone response pathway to promote mating projection formation in opaque α cells.

In this study, we provide additional evidence for the evolution of coordination between white and opaque cells during sexual mating in *C. albicans*. We demonstrate that opaque cells can induce mating-incompetent white cells to secrete pheromone (**Figure 2** and **S5**). Consistent with our data, Lin *et al.* recently reported that the expression level of *MFA1* in white **a** cells was increased ∼475 fold upon treatment with α-pheromone [42]. We note that the studies by Yi *et al.* [39] and Sanhi *et al.* [43] demonstrate that the expression of *MFA1* in white **a** cells remains unchanged in response to α-pheromone. As discussed in a recent review article [44], this discrepancy may be due to differences in laboratory growth conditions. In a system where white and opaque cells co-exist, pheromone signaling leads to the formation of a positive feedback loop, promoting the occurrence of opposite- and same-sex mating. Example scenarios of white and opaque cells co-existing, and the functional consequences of these interactions are summarized in **Figure 6A and 6B**, respectively. As shown in **Figure 6B**, opaque α cells constitutively secrete α-pheromone, which activates the pheromone response signaling pathway (Ste2-MAPK-Cph1) of white **a** cells. “Activated” white **a** cells are then induced to produce **a**-pheromone, which in turn activates the pheromone response signaling pathway (Ste3-MAPK-Cph1) and induces mating projection formation of opaque α cells. Of note, the expression of *MFA1* is extremely low, even in opaque **a** cells, although it can be enhanced by treatment of the opaque **a** cells with α-pheromone (**Figure 2** and [22]). This positive feedback loop for pheromone response is widely conserved in other yeasts. It is known that α cells can induce **a**-pheromone secretion of **a** cells in *Saccharomyces cerevisiae* [45]. Nielsen and coworkers reported that mating pheromone also triggers a positive feedback response in the fission yeast *Schizosaccharomyces pombe* [46]. This positive feedback loop for pheromone response is, therefore, a general feature in yeast species.

**Figure 7:**
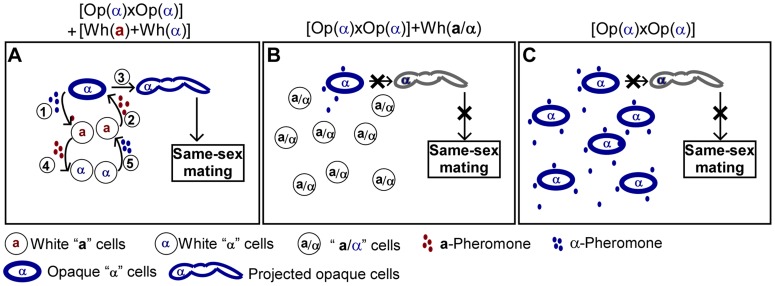
Schematic models depicting that white cells facilitate same-sex mating of opaque cells. (A) When the majority are white **a** and/or α cells, same-sex mating between the minority opaque α and α cells can occur. (1) Opaque α cells secrete α-pheromone and (2) induce the majority white **a** cells to secrete **a**-pheromone. (3) **a**-Pheromone in turn induces opaque α cells to form long mating projections and undergo same-sex mating. (4) **a**-Pheromone induces white α cells to secrete α-pheromone. (5) α-Pheromone further activates white **a** cells to secrete a-pheromone. (B) When the majority are a/α cells, the minority of opaque α cells cannot form mating projections and cannot undergo same-sex mating. Opaque α cells secrete α-pheromone constitutively, while **a**/α cells do not secrete pheromone. (C) In the absence of white **a** cells, opaque α cells cannot form mating projections and cannot undergo same-sex mating.

Since sexual mating in *C. albicans* is directed by the pheromone-mediated signaling pathway, it is perhaps not surprising that pheromone released by white cells is able to facilitate opaque cell mating by increasing the levels of extracellular pheromone. This is the case for both opposite- and same-sex mating of opaque cells (**Tables 1** and **2**). Given that the white phase is the default state, opaque cells are likely to be the minority in a natural population. In such a situation, mating between opaque cells would be rare because the concentration of pheromone produced by opaque cells would not reach the threshold required for activating the mating signaling process. Moreover, low pheromone levels do not arrest opaque cells in the G1 phase of the cell cycle [21], [47], which is a prerequisite for mating in *C. albicans*. In the presence of pheromone-secreting white **a** or α cells, the general pheromone level of the population may be increased and thus opaque cell mating could become possible. In the absence of opposite *MTL* type cells, same-sex mating is unable to occur due to the absence of the opposite mating type pheromone. In **Figure 7**, we propose a model depicting how white cells could facilitate same-sex mating of opaque cells under natural conditions. In response to α-pheromone released by opaque α cells, white **a** cells secrete **a**-pheromone and thus promote same-sex mating of opaque α cells (**Figure 7A**). In the absence of white **a** cells, same-sex mating of opaque cells could not occur (**Figure 7B and 7C**). Our experiments were performed on colonies on plates and on planktonic liquid cultures. We believe that both of these culture conditions are relevant for commensal and pathogenic lifestyles in *C. albicans*. Colonies represent an architecturally structured community, where cells exist in close proximity to one another, while the planktonic state is the state that cells exist in during a disseminated bloodstream infection. It was suggested that *C. albicans* can use different strategies to increase mating efficiency [20]. Alby and Bennett (2011) recently reported that interspecies pheromone signaling can promote same-sex mating in *C. albicans* [48]. This is interesting because *C. albicans* is often present with other microbiome members within the host, including other fungal, bacterial, and archaeal species. Therefore, to mate efficiently, opaque cells likely take advantage of a number of different strategies and may utilize a multitude of environmental signals to communicate in natural environments.

Sexual reproduction has many adaptive benefits over asexual reproduction in eukaryotic organisms. However, sexual reproduction is also an extremely costly process in terms of energy expenditure. How does *C. albicans* balance these reproductive strategies to better adapt to the changing host micro-environments, and increase its fitness during evolution over time in the host? The discovery of phenotypic switching may provide some clues to address this question. Differentiated white and opaque cells of *C. albicans* play specialized roles in these processes. Mating machinery can be simply shut down or activated through phenotypic transitions. Inducing expression of pheromone in mating-incompetent white cells and opaque **a** cells, may not only serve to save energy, but could also serve to promote sexual mating when there is a need for it. We believe that the existence of this cell type heterogeneity, creating, in a sense, a “labor division,” amongst the population, in addition to the multicellular coordination between the white and opaque cell populations, may be the primary reasons as to why this fungus is so successful at surviving and thriving in the human host as both a commensal and pathogen.

## Materials and Methods

### Culture conditions, strains and plasmids

The strains used in this study are listed in **Table S4**. All strains used are derivatives of the following independent clinical isolates: SC5314, WO-1, SZ306, and P37005. All strains used in this study are diploid. In Figures and Tables, *MTL* **a** (or “**a**”) and *MTL*α (or “α”) indicate the mating type locus is **a**/**a** (or **a**/Δ) and α/α (or “Δ/α”), respectively. Modified Lee's glucose medium [49] was used for routine culture of *C. albicans* cells and for mating projection formation and mating assays. Construction of strains:

#### SZ306u-a, SZ306u-α and SZ306w-a

To generate the strains, SZ306u-a and SZ306u-α, one allele of the *MTL* locus was deleted from strain SZ306u with the plasmid L23.14 [50]. To generate SZ306w-a, the strain SZ306w was first grown on YPmal medium (1% yeast extract, 2% peptone, 2% maltose) for FLP-mediated excision of the *SAT1/flipper* cassette. The *MTL*α allele was then deleted with the plasmid L23.14.

#### GH1602 (*wor1*Δ/Δ, a/Δ) and GH1618 (*wor1*Δ/Δ, Δ/α)

The *wor1*Δ/Δ mutant (**a**/α, GH1601 [50]) was first grown on YPmal medium for FLP-mediated excision of the *SAT1/flipper* cassette. One allele of the *MTL* locus was then deleted with the plasmid L23.14 to generate the strain GH1602 or GH1618.

#### GH1600 (MFA1p-GFP), GH1603 (*wor1*Δ/Δ MFA1p-GFP), GH1619 (*wor1*Δ/Δ MFα1p-GFP) and GH1620 (MFα1p-GFP)

To construct GFP reporter strains, SZ306a, GH1602, GH1618 and SN152α were first grown on YPmal medium for FLP-mediated excision of the *SAT1/flipper* cassette. The resulting strains were then transformed with PCR products of the *GFP-caSAT1* fragment (amplified from the template plasmid pNIM1 [51]), generating GH1600, GH1603, GH1619 and GH1620 strains. Two pairs of primers (MFA1pGFP-F and MFA1pGFP-R, MFα1pGFP-F and MFα1pGFP-R) were used for PCR amplification. Correctly GFP-labeled transformants were confirmed by PCR amplification with genomic DNA as the template and checking primers. Primers used are listed in **Table S5**.

#### GH1610, GH1611, GH1613, GH1614 and GH1615

These strains were generated by deleting the *MTL* **a** or *MTL*α in the corresponding heterozygous **a**/α strains with the plasmid L23.14 [50].

#### GH1616 (*ste3* Δ/Δ) and GH1617 (*mfα1* Δ/Δ)

Strain WUM5A was used to generate GH1616 and GH1617 mutants. To delete the first copy of *MFα1* or *STE3*, WUM5A was transformed with the fusion PCR product of the *URA3* gene flanked by *MFα1* gene or *STE3* gene 5′ and 3′ fragments [52]. The plasmid pGEM-URA3 was used to amplify *URA3* fragment [53]. The resulting strain *MFα1*/*mfα1::URA3* (or *STE3*/*ste3::URA3*) was then transformed with the PCR product of *SAT1*/flipper cassette with the primer pairs of MFα1-5DR and MFα1-3DR (or STE3-5DR and STE3-3DR). The plasmid pSFS2a [54] was used as a PCR template. The mutants were verified by PCR using several primer sets.

#### GH1605 and GH1606

These strains were generated by growing SN152 and SN152a on the SD medium containing 5-fluoroorotic acid (5-FOA) and uridine.

#### GH1607 and GH1608

To construct the *wor1*Δ/Δ mutant in SN152 (**a**/α), the two alleles of *WOR1* were sequentially deleted using the fusion PCR strategy [52]. Primers used are listed in **Table S5**. To generate GH1608, the *MTL*α allele was disrupted in GH1607 with the plasmid L23.14 [50].

### Assay of pheromone-induced mating projection formation

A 14-mer α-pheromone peptide (GFRLTNFGYFEPGK) of *C. albicans* was chemically synthesized. Cells of *C. albicans* were first grown in liquid media at 25°C for 36 hours to stationary phase and then inoculated into fresh Lee's glucose medium (1×10^7^ cells/ml) for pheromone treatment assays. α-Pheromone peptide was added to the cultures every two hours after inoculation over an eight-hour period of growth. The final concentration of α-pheromone in the cultures was 8×10^−6^ M.

### Assay of white cells induced-mating projection formation

4×10^6^ opaque cells were mixed with equal number of white cells of different background. The mixtures were spotted onto Lee's glucose medium plates and cultured at 25°C in air. After a 24-hour incubation period, cells were examined under a microscope and the ratio of “projected” opaque cells in each mixture was calculated.

### Mating assays

Opposite-sex mating assays were performed according to our previous publication with modifications [50]. Briefly, 4.8×10^6^ of “helper” white cells of different background were added to the mating mixture (1×10^4^ opaque **a** cells plus 3.2×10^6^ opaque α cells). The mating mixtures were spotted onto Lee's glucose medium plates and cultured at 25°C for two to five days as indicated in the table legends. The mating mixtures were replated onto SD-histidine-uridine, SD-arginine-uridine and SD-uridine-arginine-histidine media for prototrophic selection growth. All the “helper” strains are *ura3*Δ/Δ mutants and could not grow on media without uridine.

The opposite-sex mating assay of “**a**-op (*mfa1Δ/Δ*)×α-op” cross: 9.6×10^7^ white cells of “helper” strains (60%) were mixed with 3.2×10^7^ opaque **a** cells of GH1013 (**a**/**a**, *ura3Δ/Δ, mfa1Δ/Δ*, 20%) and 3.2×10^7^ opaque cells of GH1349 (α/α, *arg4Δ/Δ*, 20%). The mating mixtures were spotted onto Lee's glucose medium plates and cultured at 25°C for five days. Then, mixed cells were replated onto SD-uridine and SD-arginine-uridine media for prototrophic selection growth.

The same-sex mating assay for the “α-op×α-op” cross: 9.6×10^7^ white cells of “helper” strains (60%) were mixed with 3.2×10^7^ opaque cells of SZ306α (*Δ*/α, *ura3Δ/Δ*, 20%) and 3.2×10^7^ opaque cells of GH1349 (α/α, *arg4Δ/Δ*, 20%). The mating mixtures were spotted onto Lee's glucose medium plates and cultured at 25°C for five days. Then, mixed cells were replated onto SD-uridine and SD-arginine-uridine media for prototrophic selection growth. PCR of the *MTL* **a**1 and α2 was used to confirm the tetraploid colonies of “α-op×α-op” fusion and to exclude possible tetraploid α/**a** colonies due to the low-frequency of the “α-op×**a**-wh” fusion.

The same-sex mating assay for the “**a**-op×**a**-op” cross (**Figure S5C**). Opaque cells of GH1013h (**a**/**a**, *his1*Δ/Δ) were first grown in liquid Lee's glucose medium at 25°C for 24 h. Cells were then harvested and resuspended in fresh Lee's glucose medium (2×10^8^ cells/ml) containing 10^−4^ M of α-pheromone peptide and incubated at 25°C for an eight-hour period of growth. 9.6×10^7^ white cells of “helper” strains (60%) were mixed with 3.2×10^7^ α-pheromone-treated opaque cells of GH1013h (**a**/**a**, *his1*Δ/Δ, 20%) and 3.2×10^7^ opaque cells of SZ306u-a (**a**/Δ, *ura3Δ/Δ*, 20%). The mixture of opaque “**a**” cells (GH1013h) and opaque “**a**” cells (SZ306u-a) served as a negative control. The mating mixtures were spotted onto Lee's glucose medium plates and cultured at 25°C for four days. Then, mixed cells were replated onto SD-uridine and SD-histidine-uridine media for prototrophic selection growth. PCR of the *MTL* **a**1 and α2 was used to verify the tetraploid colonies of “**a**-op×**a**-op” fusion and to exclude possible tetraploid **a**/α colonies due to the low-frequency of “**a**-op×α-wh” mating.

### RNA extraction, RNA-Seq, and quantitative real-time PCR (Q-RT-PCR) assays

Cells were first grown in Lee's glucose liquid medium at 25°C for 24 hours and inoculated into fresh Lee's glucose medium (1×10^7^ cells/ml). α-Pheromone peptide was added to the cultures every 8 hours over a 24 hours period. The final concentration of α-pheromone in the culture was 1.6×10^−5^ M. Cells were then collected and total RNA was extracted for RNA-Seq analysis and quantitative PCR assays. RNA-Seq analysis was performed by the company BGI-Shenzhen according to the company's protocol [55]. Approximately 10 million (M) reads were obtained by sequencing each library. The library products were sequenced using the Illumina HiSeq 2000. Illumina OLB_1.9.4 software was used for base-calling. The raw reads were filtered by removing the adapter and low quality reads (the percentage of low quality bases with a quality value ≤5,>50% in a read). Clean reads were mapped to the genome of *C. albicans* SC5314 using SOAP aligner/soap2 software (version 2.21) [56]. The more complete and detailed RNA-seq dataset has been deposited into the NCBI Gene Expression Omnibus (GEO) portal (Accession number: GSE56039). Q-RT-PCR assays were performed to verify the relative gene expression levels of pheromone-treated and untreated samples. Quantitative PCR was performed according to our previous publication with modifications [57]. Briefly, 0.6 µg of total RNA per sample were used to synthesize cDNA with RevertAid H Minus Reverse Transcriptase (Thermo Scientific). Quantification of transcripts was performed in Bio-Rad CFX96 real-time PCR detection system using SYBR green. The signal from each experimental sample was normalized to expression of the *ACT1* gene.

### Animal experiments

Skin infection assays were performed as described previously [57]. Newborn ICR mice (2 to 4 days old) were used. *In vivo* skin mating assay of the “**a**-op×α-op” cross: 1.2×10^8^ white cells of “helper” strains (∼60%) were mixed with 2.5×10^5^ opaque cells of GH1013h (**a**/**a**, *his1*Δ/Δ) and 8×10^7^ opaque cells of GH1349 (α/α, *arg4*Δ/Δ, ∼40%). The mixture of opaque “**a**” cells (GH1013h) and opaque “α” cells (GH1349) served as a control. The mating mixtures were spotted onto the skin on the back of a newborn mouse. After water evaporated, a small sterile filter paper with First Aid tape was used to cover the area of the fungal spot. After two days of infection, *C. albicans* cells colonized on mouse skin were washed with PBS and plated onto SD-arginine-histidine-uridine and SD-histidine-uridine media for prototrophic selection growth.

SEM assays. 1×10^7^ white “helper” cells were mixed with 1×10^7^ opaque “α” cells. The mixtures were used for the skin infection. The infection method was similar to that of the quantitative mating assay. After 24 h of infection, the infected skin areas with *C. albicans* cells were excised for SEM assays according to our previous protocols [57].

### Ethics statement

All animal experiments were performed according to the guidelines approved by the Animal Care and Use Committee of the Institute of Microbiology, Chinese Academy of Sciences. The present study was approved by the Committee.

## Supporting Information

Figure S1 White **a** cells induce mating projection formation in opaque α cells in liquid Lee's medium. Scale bar, 10 µm. (A) Cellular images of single strain cultures. (B) Cellular images of mixed cultures. 4×10^6^ opaque α cells (GH1349) were mixed with 4×10^6^ white cells of SZ306a or GH1248 (*wor1*Δ/Δ) as indicated. The mixtures were cultured in Lee's glucose medium at 25°C for 24 hours. Cellular images and percentages of opaque α cells (GH1349) with mating projections are shown. Cells with at least one mating projection were counted. The mixture of opaque α cells (GH1349) and opaque **a** cells (GH1012) served as a positive control. The mixture of opaque α cells (GH1349) and SZ306 (**a**/α) served as a negative control. NA, not available. W (or wh), white; O (or op), opaque; P, mating projection.
(TIF)

Figure S2 Effect of the ratio of white **a** cells to opaque α cells on the induction of mating projections. White **a** cells of strains SZ306a, *wor1*Δ/Δ (GH1248) or *wor2*Δ/Δ (MMY627) were mixed with opaque α cells (GH1349) at different ratios (as indicated). The mixtures were spotted onto Lee's glucose medium and incubated at 25°C for 24 hours. Cellular images and percentages of opaque α cells (GH1349) with mating projections are shown. P, mating projection. Scale bar, 10 µm.
(TIF)

Figure S3 4′-6-diamidino-2-phenylindole (DAPI)-DNA staining assays. Cells were fixed with 3.7% formaldehyde for 1 hour and washed with 1× PBS before staining. To stain nuclear DNA, cells were then incubated in PBS with 1 µg/ml DAPI for 10 min at room temperature. Scale bar, 10 µm.
(TIF)

Figure S4 Expression of *MFα1* in opaque α cells. An MFα1p-GFP reporter strain was constructed in SN152α. Cells were cultured in Lee's glucose medium overnight at 25°C and re-inoculated into fresh medium for 6 to 48 hours. DIC and GFP images at different time points are shown. Scale bar, 10 µm.
(TIF)

Figure S5 White α cells express α-pheromone and facilitate sexual mating of opaque **a** cells. (A) Expression of *MFα1* in white α cells. White cells of the MFα1p-GFP reporter strain (Figure S4) were mixed with opaque **a**, α, or **a** and α cells. (B) Expression of *MFα1* in opaque α cells in single or mixed cultures. (C) White α cells can facilitate same-sex mating of opaque **a** cells. To induce the expression of *MFA1*, opaque cells of GH1013h were first treated with α–pheromone. Cross: GH1013h (4×10^7^ of opaque **a** cells)×SZ306u-a (4×10^7^ of opaque **a** cells). White cells (1.2×10^8^) of **a**/α or α strains were mixed with opaque cells of the mating cross strains. The mixtures were spotted onto Lee's glucose medium and cultured at 25°C in air for 4 days. Detailed methods are provided in the Materials and methods sections. * No white “helper”: the pure opaque cell mixture (GH1013h×SZ306u-a) was also used as a control.
(TIF)

Figure S6 *MFα1* in opaque α cells is required for the induction of *MFA1* expression in white **a** cells. Two reporter strains: GH1600 (MFA1p-GFP) and GH1603 (*wor1*Δ/Δ MFA1p-GFP). 4×10^6^ opaque α cells of the WT and *mfα1*Δ/Δ mutant were mixed with 4×10^6^ white **a** cells (reporter strain). The mixtures were spotted onto Lee's glucose medium and incubated at 25°C for 24 hours. Expression of GFP proteins in white cells of the reporter strains was examined with a fluorescence microscope (A). Images of single strain cultures (white cells of the reporter strains) are shown in (B). wh, white; op, opaque. BF, bright field. Scale bar, 10 µm.
(TIF)

Figure S7 Relative expression levels of *MFA1* (A), *STE2* (B), and *STE3* (C), in white and opaque cells. Pheromone treatment and Q-RT-PCR assays were performed as described in the Materials and Methods section. The value of the expression level of each gene in the WT (**a**/α) strain was set as “1”.
(TIF)

Figure S8 Pheromone-response genes in white cells. (A) Pheromone-up-regulated (75 genes) and down-regulated (124 genes); two-fold threshold cutoff. (B) Functional category of Pheromone-regulated genes. (C) Verification of the relative expression levels of eight pheromone-regulated genes by Q-RT-PCR assays. The *wor1Δ/Δ* mutant GH1602 was used for RNA-Seq and Q-RT-PCR analysis. The value of the expression level of each gene in pheromone-untreated cells was set as “1”.
(TIF)

Table S1 White a cells induce mating projection formation in opaque α cells but mate poorly with opaque α cells. 1×10^6^ white **a** cells of the WT or *wor1*Δ/Δ mutant were mixed with 1×10^6^ opaque α cells. The mixtures were spotted onto Lee's glucose medium plates and cultured at 25°C for 48 hours. Mixed cells were replated onto SD medium for prototrophic selection. Mating efficiency and percentages of cells with mating projections for each mixed culture are shown. <, indicates that no progeny colonies (mating efficiency) or no opaque α cells with mating projections were observed. The cross of opaque **a** cells (GH1012) and opaque α cells (GH1349) served as a positive control. The cross of white **a** cells (SN152a) and white α cells (SZ306α) served as a negative control. N.A, not analyzed.
(DOC)

Table S2 White “a” cells facilitate opposite-sex mating of opaque cells in a mouse skin infection model. Newborn ICR mice (2 to 4 days old) were used for infection. “**a**-op xα-op” mating cross: 1.2×10^8^ white cells of “helper” strains (∼60%) were mixed with 2.5×10^5^ opaque cells of GH1013h (**a**/**a**, *his1*Δ/Δ) and 8×10^7^ opaque cells of GH1349 (α/α, *arg4*Δ/Δ). The mixture of opaque “**a**” cells (GH1013h) and opaque “α” cells (GH1349) served as the control (no “helper” white cells).
(DOC)

Table S3 Functional description and categorization of pheromone-regulated genes identified by RNA-Seq analysis.
(XLS)

Table S4 Strains used in this study.
(DOC)

Table S5 Primers used in this study.
(DOCX)

## References

[1] Barton NH, Charlesworth B (1998) Why sex and recombination? Science 281: 1986–1990. 9748151

[2] Otto SP, Lenormand T (2002) Resolving the paradox of sex and recombination. Nat Rev Genet 3: 252–261. 11967550 10.1038/nrg761

[3] Lee SC, Ni M, Li W, Shertz C, Heitman J (2010) The evolution of sex: a perspective from the fungal kingdom. Microbiol Mol Biol Rev 74: 298–340. 20508251 10.1128/MMBR.00005-10 PMC2884414

[4] Ni M, Feretzaki M, Sun S, Wang X, Heitman J (2011) Sex in fungi. Annu Rev Genet 45: 405–430. 21942368 10.1146/annurev-genet-110410-132536 PMC3310392

[5] Johnson A (2003) The biology of mating in Candida albicans. Nat Rev Microbiol 1: 106–116. 15035040 10.1038/nrmicro752

[6] Heitman J (2006) Sexual reproduction and the evolution of microbial pathogens. Curr Biol 16: R711–725. 16950098 10.1016/j.cub.2006.07.064

[7] Ene IV, Bennett RJ (2014) The cryptic sexual strategies of human fungal pathogens. Nat Rev Microbiol 12: 239–251. 24625892 10.1038/nrmicro3236 PMC4102497

[8] Hull CM, Raisner RM, Johnson AD (2000) Evidence for mating of the “asexual” yeast Candida albicans in a mammalian host. Science 289: 307–310. 10894780 10.1126/science.289.5477.307

[9] Magee BB, Magee PT (2000) Induction of mating in Candida albicans by construction of MTLa and MTLalpha strains. Science 289: 310–313. 10894781 10.1126/science.289.5477.310

[10] Alby K, Schaefer D, Bennett RJ (2009) Homothallic and heterothallic mating in the opportunistic pathogen Candida albicans. Nature 460: 890–893. 19675652 10.1038/nature08252 PMC2866515

[11] Lockhart SR, Pujol C, Daniels KJ, Miller MG, Johnson AD, et al (2002) In Candida albicans, white-opaque switchers are homozygous for mating type. Genetics 162: 737–745. 12399384 10.1093/genetics/162.2.737 PMC1462282

[12] Slutsky B, Staebell M, Anderson J, Risen L, Pfaller M, et al (1987) “White-opaque transition”: a second high-frequency switching system in Candida albicans. J Bacteriol 169: 189–197. 3539914 10.1128/jb.169.1.189-197.1987 PMC211752

[13] Miller MG, Johnson AD (2002) White-opaque switching in Candida albicans is controlled by mating-type locus homeodomain proteins and allows efficient mating. Cell 110: 293–302. 12176317 10.1016/s0092-8674(02)00837-1

[14] Anderson JM, Soll DR (1987) Unique phenotype of opaque cells in the white-opaque transition of Candida albicans. J Bacteriol 169: 5579–5588. 3316187 10.1128/jb.169.12.5579-5588.1987 PMC213989

[15] Lan CY, Newport G, Murillo LA, Jones T, Scherer S, et al (2002) Metabolic specialization associated with phenotypic switching in Candidaalbicans. Proc Natl Acad Sci U S A 99: 14907–14912. 12397174 10.1073/pnas.232566499 PMC137518

[16] Soll DR (2009) Why does Candida albicans switch? FEMS Yeast Res 9: 973–989. 19744246 10.1111/j.1567-1364.2009.00562.x

[17] Huang G (2012) Regulation of phenotypic transitions in the fungal pathogen Candida albicans. Virulence 3: 251–61 doi: 10.4161/viru.20010 22546903 10.4161/viru.20010 PMC3442837

[18] Lohse MB, Johnson AD (2009) White-opaque switching in Candida albicans. Curr Opin Microbiol 12: 650–654. 19853498 10.1016/j.mib.2009.09.010 PMC2812476

[19] Daniels KJ, Srikantha T, Lockhart SR, Pujol C, Soll DR (2006) Opaque cells signal white cells to form biofilms in Candida albicans. EMBO J 25: 2240–2252. 16628217 10.1038/sj.emboj.7601099 PMC1462973

[20] Park YN, Daniels KJ, Pujol C, Srikantha T, Soll DR (2013) Candida albicans forms a specialized “sexual” as well as “pathogenic” biofilm. Eukaryot Cell 12: 1120–1131. 23771904 10.1128/EC.00112-13 PMC3754541

[21] Bennett RJ, Johnson AD (2006) The role of nutrient regulation and the Gpa2 protein in the mating pheromone response of C. albicans. Mol Microbiol 62: 100–119. 16987174 10.1111/j.1365-2958.2006.05367.x

[22] Dignard D, El-Naggar AL, Logue ME, Butler G, Whiteway M (2007) Identification and characterization of MFA1, the gene encoding Candida albicans a-factor pheromone. Eukaryot Cell 6: 487–494. 17209123 10.1128/EC.00387-06 PMC1828930

[23] Fraser JA, Giles SS, Wenink EC, Geunes-Boyer SG, Wright JR, et al (2005) Same-sex mating and the origin of the Vancouver Island Cryptococcus gattii outbreak. Nature 437: 1360–1364. 16222245 10.1038/nature04220

[24] Lin X, Hull CM, Heitman J (2005) Sexual reproduction between partners of the same mating type in Cryptococcus neoformans. Nature 434: 1017–1021. 15846346 10.1038/nature03448

[25] Huang G, Wang H, Chou S, Nie X, Chen J, et al (2006) Bistable expression of WOR1, a master regulator of white-opaque switching in Candida albicans. Proc Natl Acad Sci U S A 103: 12813–12818. 16905649 10.1073/pnas.0605270103 PMC1540355

[26] Srikantha T, Borneman AR, Daniels KJ, Pujol C, Wu W, et al (2006) TOS9 regulates white-opaque switching in Candida albicans. Eukaryot Cell 5: 1674–1687. 16950924 10.1128/EC.00252-06 PMC1595353

[27] Zordan RE, Galgoczy DJ, Johnson AD (2006) Epigenetic properties of white-opaque switching in Candida albicans are based on a self-sustaining transcriptional feedback loop. Proc Natl Acad Sci U S A 103: 12807–12812. 16899543 10.1073/pnas.0605138103 PMC1535343

[28] Bennett RJ, Uhl MA, Miller MG, Johnson AD (2003) Identification and characterization of a Candida albicans mating pheromone. Mol Cell Biol 23: 8189–8201. 14585977 10.1128/MCB.23.22.8189-8201.2003 PMC262406

[29] Lockhart SR, Zhao R, Daniels KJ, Soll DR (2003) Alpha-pheromone-induced “shmooing” and gene regulation require white-opaque switching during Candida albicans mating. Eukaryot Cell 2: 847–855. 14555467 10.1128/EC.2.5.847-855.2003 PMC219372

[30] Panwar SL, Legrand M, Dignard D, Whiteway M, Magee PT (2003) MFalpha1, the gene encoding the alpha mating pheromone of Candida albicans. Eukaryot Cell 2: 1350–1360. 14665468 10.1128/EC.2.6.1350-1360.2003 PMC326654

[31] Madhani HD, Fink GR (1997) Combinatorial control required for the specificity of yeast MAPK signaling. Science 275: 1314–1317. 9036858 10.1126/science.275.5304.1314

[32] Elion EA (2000) Pheromone response, mating and cell biology. Curr Opin Microbiol 3: 573–581. 11121776 10.1016/s1369-5274(00)00143-0

[33] Kohler JR, Fink GR (1996) Candida albicans strains heterozygous and homozygous for mutations in mitogen-activated protein kinase signaling components have defects in hyphal development. Proc Natl Acad Sci U S A 93: 13223–13228. 8917572 10.1073/pnas.93.23.13223 PMC24074

[34] Wang P, Heitman J (1999) Signal transduction cascades regulating mating, filamentation, and virulence in Cryptococcus neoformans. Curr Opin Microbiol 2: 358–362. 10458985 10.1016/S1369-5274(99)80063-0

[35] Pan X, Harashima T, Heitman J (2000) Signal transduction cascades regulating pseudohyphal differentiation of Saccharomyces cerevisiae. Curr Opin Microbiol 3: 567–572. 11121775 10.1016/s1369-5274(00)00142-9

[36] Whiteway M, Bachewich C (2007) Morphogenesis in Candida albicans. Annu Rev Microbiol 61: 529–553. 17506678 10.1146/annurev.micro.61.080706.093341 PMC4452225

[37] Liu H, Kohler J, Fink GR (1994) Suppression of hyphal formation in Candida albicans by mutation of a STE12 homolog. Science 266: 1723–1726. 7992058 10.1126/science.7992058

[38] Chen J, Lane S, Liu H (2002) A conserved mitogen-activated protein kinase pathway is required for mating in Candida albicans. Mol Microbiol 46: 1335–1344. 12453219 10.1046/j.1365-2958.2002.03249.x

[39] Yi S, Sahni N, Daniels KJ, Pujol C, Srikantha T, et al (2008) The same receptor, G protein, and mitogen-activated protein kinase pathway activate different downstream regulators in the alternative white and opaque pheromone responses of Candida albicans. Mol Biol Cell 19: 957–970. 18162580 10.1091/mbc.E07-07-0688 PMC2262975

[40] Yi S, Sahni N, Daniels KJ, Lu KL, Srikantha T, et al (2011) Alternative mating type configurations (a/alpha versus a/a or alpha/alpha) of Candida albicans result in alternative biofilms regulated by different pathways. PLoS Biol 9: e1001117. 21829325 10.1371/journal.pbio.1001117 PMC3149048

[41] Magee BB, Legrand M, Alarco AM, Raymond M, Magee PT (2002) Many of the genes required for mating in Saccharomyces cerevisiae are also required for mating in Candida albicans. Mol Microbiol 46: 1345–1351. 12453220 10.1046/j.1365-2958.2002.03263.x

[42] Lin CH, Kabrawala S, Fox EP, Nobile CJ, Johnson AD, et al (2013) Genetic control of conventional and pheromone-stimulated biofilm formation in Candida albicans. PLoS Pathog 9: e1003305. 23637598 10.1371/journal.ppat.1003305 PMC3630098

[43] Sahni N, Yi S, Daniels KJ, Srikantha T, Pujol C, et al (2009) Genes selectively up-regulated by pheromone in white cells are involved in biofilm formation in Candida albicans. PLoS Pathog 5: e1000601. 19798425 10.1371/journal.ppat.1000601 PMC2745568

[44] Soll DR (2014) The role of phenotypic switching in the basic biology and pathogenesis of Candida albicans. J Oral Microbiol 6: 6 doi: 10.3402/jom.v6.22993 10.3402/jom.v6.22993 PMC3895265 24455104

[45] Strazdis JR, MacKay VL (1983) Induction of yeast mating pheromone a-factor by alpha cells. Nature 305: 543–545. 6353246 10.1038/305543a0

[46] Nielsen O, Egel R (1990) The pat1 protein kinase controls transcription of the mating-type genes in fission yeast. EMBO J 9: 1401–1406. 2328719 10.1002/j.1460-2075.1990.tb08255.x PMC551826

[47] Zhao R, Daniels KJ, Lockhart SR, Yeater KM, Hoyer LL, et al (2005) Unique aspects of gene expression during Candida albicans mating and possible G(1) dependency. Eukaryot Cell 4: 1175–1190. 16002644 10.1128/EC.4.7.1175-1190.2005 PMC1168966

[48] Alby K, Bennett RJ (2011) Interspecies pheromone signaling promotes biofilm formation and same-sex mating in Candida albicans. Proc Natl Acad Sci U S A 108: 2510–2515. 21262815 10.1073/pnas.1017234108 PMC3038756

[49] Huang G, Yi S, Sahni N, Daniels KJ, Srikantha T, et al (2010) N-acetylglucosamine induces white to opaque switching, a mating prerequisite in Candida albicans. PLoS Pathog 6: e1000806. 20300604 10.1371/journal.ppat.1000806 PMC2837409

[50] Xie J, Tao L, Nobile CJ, Tong Y, Guan G, et al (2013) White-opaque switching in natural MTLa/alpha isolates of Candida albicans: evolutionary implications for roles in host adaptation, pathogenesis, and sex. PLoS Biol 11: e1001525. 23555196 10.1371/journal.pbio.1001525 PMC3608550

[51] Park YN, Morschhauser J (2005) Tetracycline-inducible gene expression and gene deletion in Candida albicans. Eukaryot Cell 4: 1328–1342. 16087738 10.1128/EC.4.8.1328-1342.2005 PMC1214539

[52] Noble SM, Johnson AD (2005) Strains and strategies for large-scale gene deletion studies of the diploid human fungal pathogen Candida albicans. Eukaryot Cell 4: 298–309. 15701792 10.1128/EC.4.2.298-309.2005 PMC549318

[53] Wilson RB, Davis D, Mitchell AP (1999) Rapid hypothesis testing with Candida albicans through gene disruption with short homology regions. J Bacteriol 181: 1868–1874. 10074081 10.1128/jb.181.6.1868-1874.1999 PMC93587

[54] Reuss O, Vik A, Kolter R, Morschhauser J (2004) The SAT1 flipper, an optimized tool for gene disruption in Candida albicans. Gene 341: 119–127. 15474295 10.1016/j.gene.2004.06.021

[55] Wang B, Guo G, Wang C, Lin Y, Wang X, et al (2010) Survey of the transcriptome of Aspergillus oryzae via massively parallel mRNA sequencing. Nucleic Acids Res 38: 5075–5087. 20392818 10.1093/nar/gkq256 PMC2926611

[56] Li R, Yu C, Li Y, Lam TW, Yiu SM, et al (2009) SOAP2: an improved ultrafast tool for short read alignment. Bioinformatics 25: 1966–1967. 19497933 10.1093/bioinformatics/btp336

[57] Tao L, Du H, Guan G, Dai Y, Nobile CJ, et al (2014) Discovery of a “white-gray-opaque” tristable phenotypic switching system in candida albicans: roles of non-genetic diversity in host adaptation. PLoS Biol 12: e1001830. 24691005 10.1371/journal.pbio.1001830 PMC3972085

