# Supplementary figures and images for: White Cells Facilitate Opposite- and Same-Sex Mating of Opaque Cells in Candida albicans
Source: PLoS Genet. 2014 Oct 16;10(10):e1004737. doi: 10.1371/journal.pgen.1004737 (PMC4199524; doi:10.1371/journal.pgen.1004737)

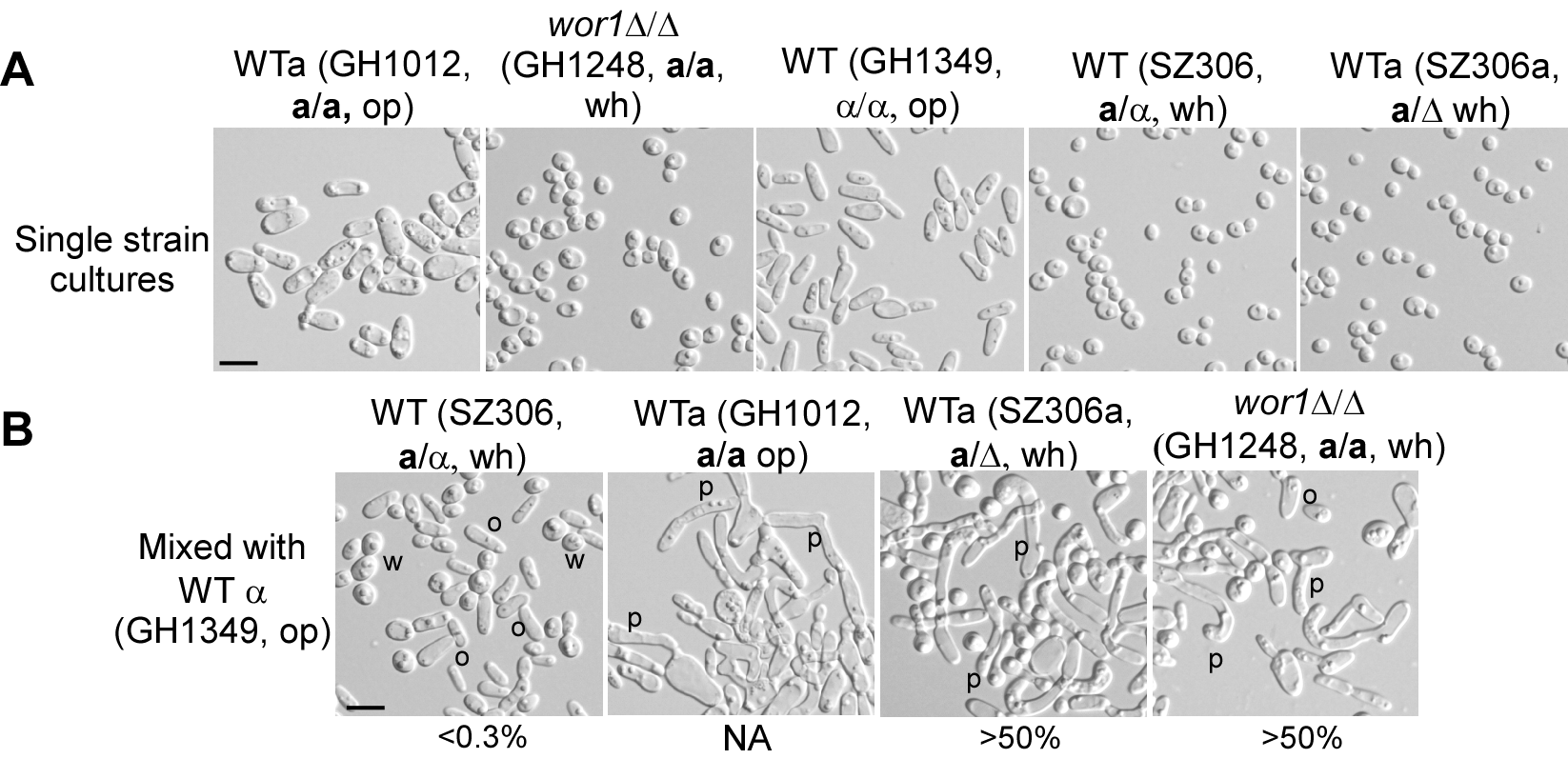

Supplement: Figure S1 — White a cells induce mating projection formation in opaque α cells in liquid Lee's medium. Scale bar, 10 µm. (A) Cellular images of single strain cultures. (B) Cellular images of mixed cultures. 4×10^6^ opaque α cells (GH1349) were mixed with 4×10^6^ white cells of SZ306a or GH1248 (wor1Δ/Δ) as indicated. The mixtures were cultured in Lee's glucose medium at 25°C for 24 hours. Cellular images and percentages of opaque α cells (GH1349) with mating projections are shown. Cells with at least one mating projection were counted. The mixture of opaque α cells (GH1349) and opaque a cells (GH1012) served as a positive control. The mixture of opaque α cells (GH1349) and SZ306 (a/α) served as a negative control. NA, not available. W (or wh), white; O (or op), opaque; P, mating projection. (TIF) [file pgen.1004737.s001.tif]

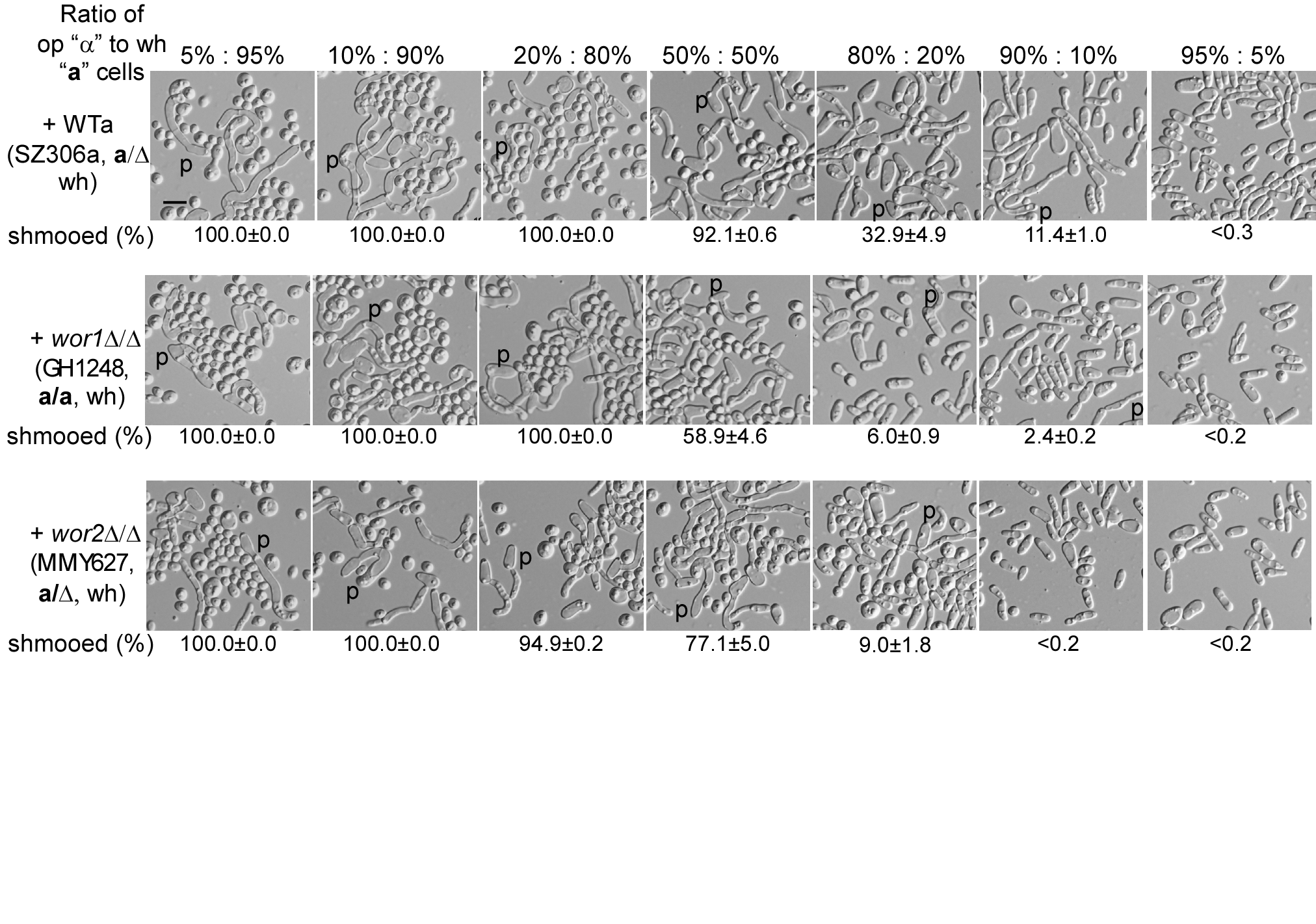

Supplement: Figure S2 — Effect of the ratio of white a cells to opaque α cells on the induction of mating projections. White a cells of strains SZ306a, wor1Δ/Δ (GH1248) or wor2Δ/Δ (MMY627) were mixed with opaque α cells (GH1349) at different ratios (as indicated). The mixtures were spotted onto Lee's glucose medium and incubated at 25°C for 24 hours. Cellular images and percentages of opaque α cells (GH1349) with mating projections are shown. P, mating projection. Scale bar, 10 µm. (TIF) [file pgen.1004737.s002.tif]

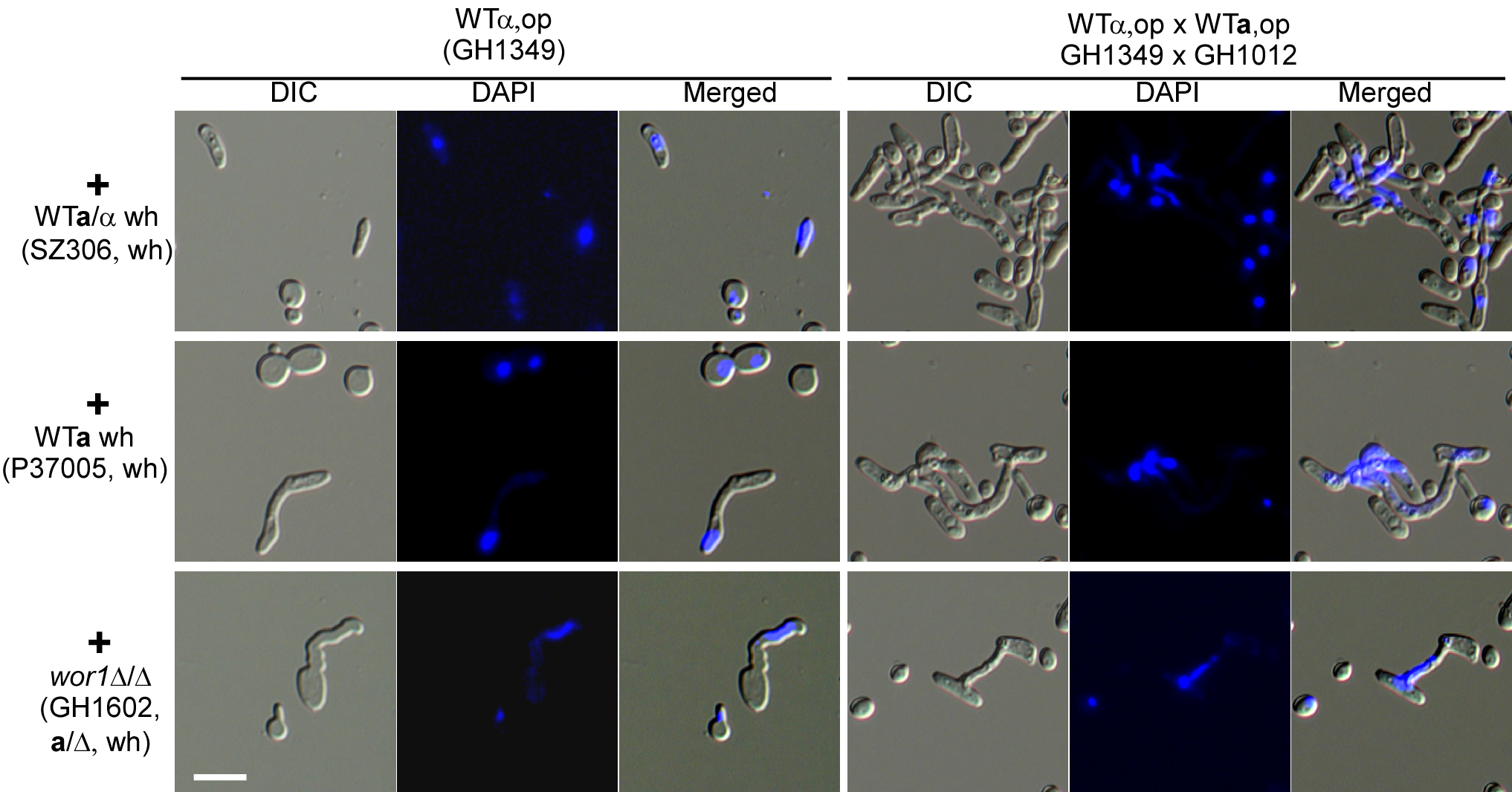

Supplement: Figure S3 — 4′-6-diamidino-2-phenylindole (DAPI)-DNA staining assays. Cells were fixed with 3.7% formaldehyde for 1 hour and washed with 1× PBS before staining. To stain nuclear DNA, cells were then incubated in PBS with 1 µg/ml DAPI for 10 min at room temperature. Scale bar, 10 µm. (TIF) [file pgen.1004737.s003.tif]

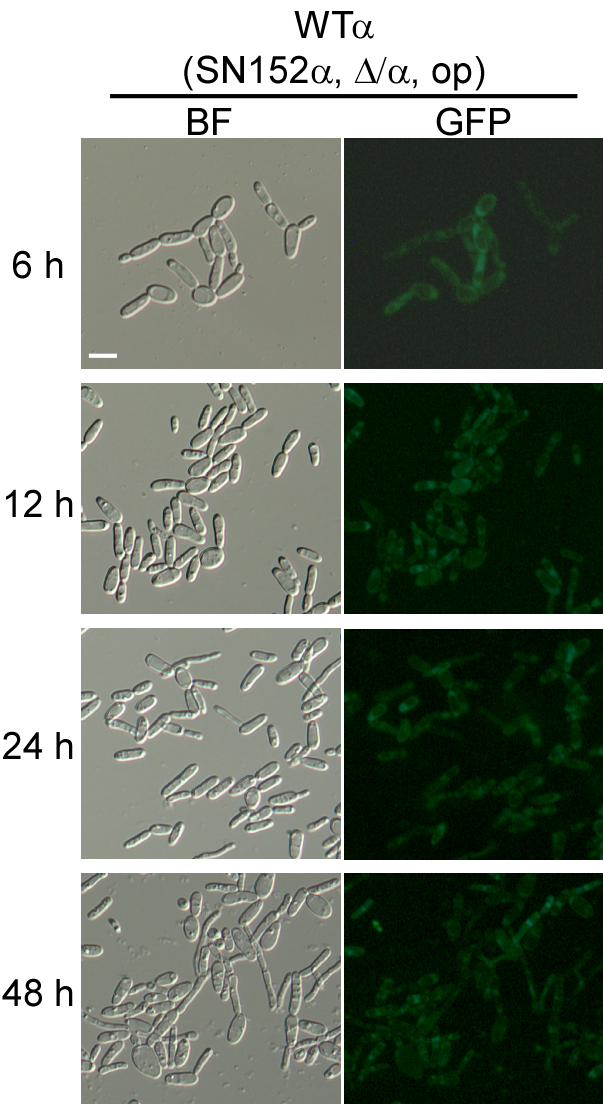

Supplement: Figure S4 — Expression of MFα1 in opaque α cells. An MFα1p-GFP reporter strain was constructed in SN152α. Cells were cultured in Lee's glucose medium overnight at 25°C and re-inoculated into fresh medium for 6 to 48 hours. DIC and GFP images at different time points are shown. Scale bar, 10 µm. (TIF) [file pgen.1004737.s004.tif]

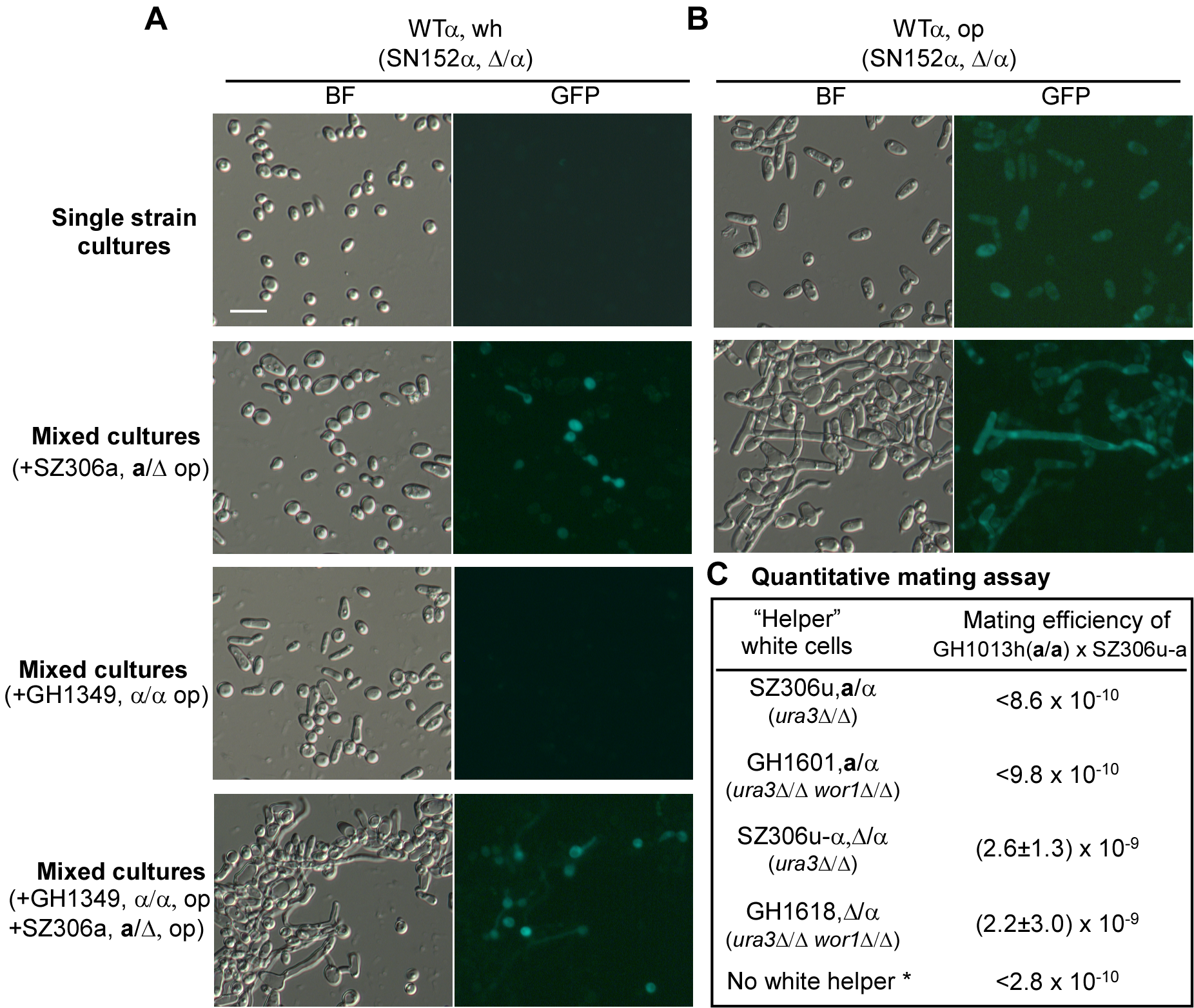

Supplement: Figure S5 — White α cells express α-pheromone and facilitate sexual mating of opaque a cells. (A) Expression of MFα1 in white α cells. White cells of the MFα1p-GFP reporter strain (Figure S4) were mixed with opaque a, α, or a and α cells. (B) Expression of MFα1 in opaque α cells in single or mixed cultures. (C) White α cells can facilitate same-sex mating of opaque a cells. To induce the expression of MFA1, opaque cells of GH1013h were first treated with α–pheromone. Cross: GH1013h (4×10^7^ of opaque a cells)×SZ306u-a (4×10^7^ of opaque a cells). White cells (1.2×10^8^) of a/α or α strains were mixed with opaque cells of the mating cross strains. The mixtures were spotted onto Lee's glucose medium and cultured at 25°C in air for 4 days. Detailed methods are provided in the Materials and methods sections. * No white “helper”: the pure opaque cell mixture (GH1013h×SZ306u-a) was also used as a control. (TIF) [file pgen.1004737.s005.tif]

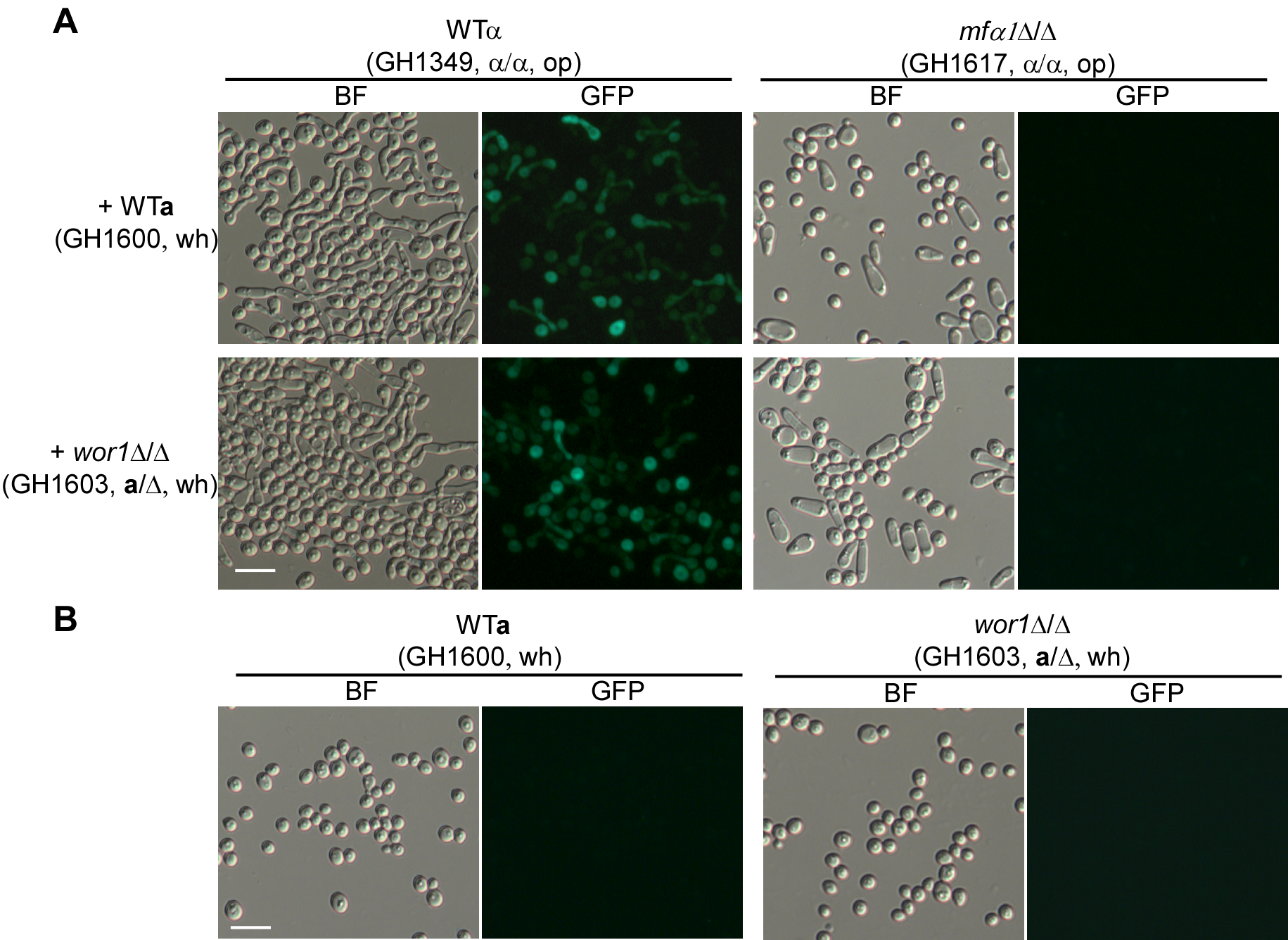

Supplement: Figure S6 — MFα1 in opaque α cells is required for the induction of MFA1 expression in white a cells. Two reporter strains: GH1600 (MFA1p-GFP) and GH1603 (wor1Δ/Δ MFA1p-GFP). 4×10^6^ opaque α cells of the WT and mfα1Δ/Δ mutant were mixed with 4×10^6^ white a cells (reporter strain). The mixtures were spotted onto Lee's glucose medium and incubated at 25°C for 24 hours. Expression of GFP proteins in white cells of the reporter strains was examined with a fluorescence microscope (A). Images of single strain cultures (white cells of the reporter strains) are shown in (B). wh, white; op, opaque. BF, bright field. Scale bar, 10 µm. (TIF) [file pgen.1004737.s006.tif]

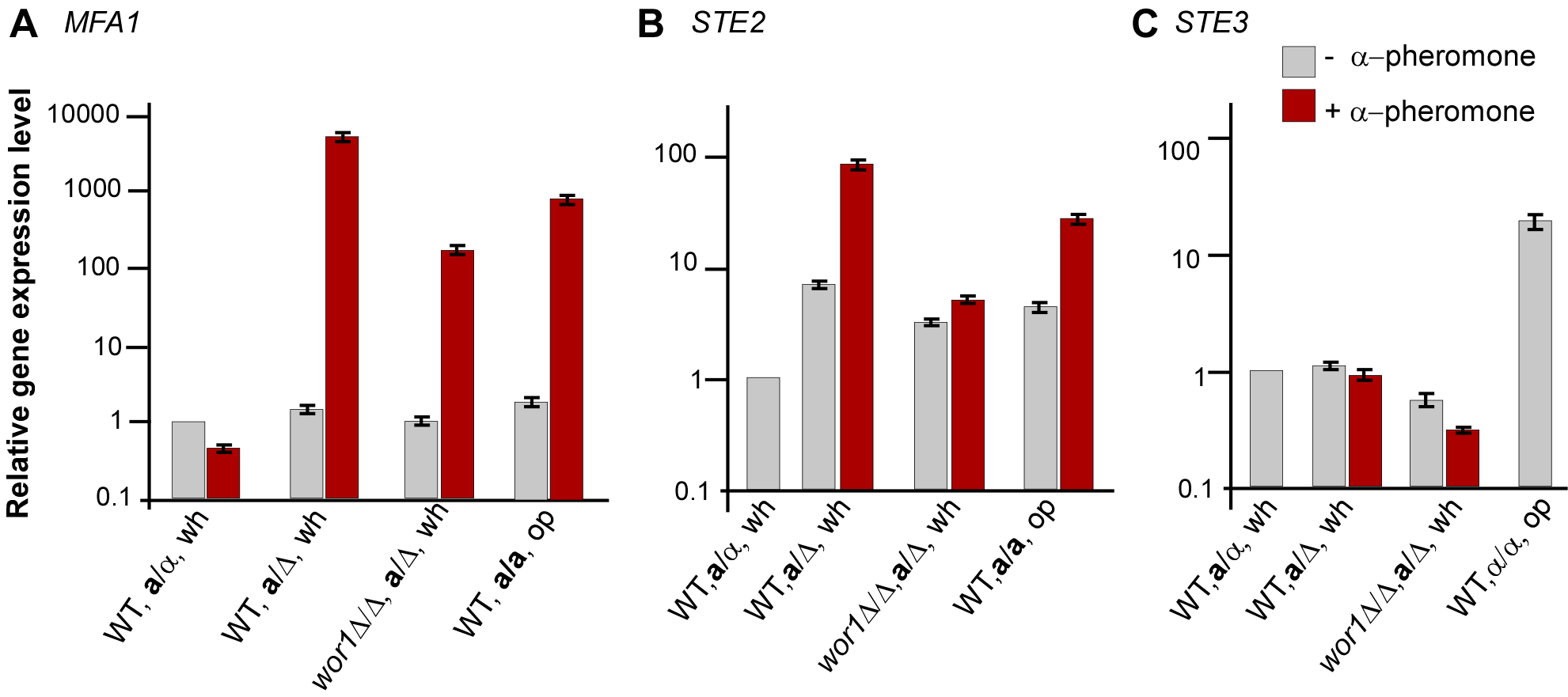

Supplement: Figure S7 — Relative expression levels of MFA1 (A), STE2 (B), and STE3 (C), in white and opaque cells. Pheromone treatment and Q-RT-PCR assays were performed as described in the Materials and Methods section. The value of the expression level of each gene in the WT (a/α) strain was set as “1”. (TIF) [file pgen.1004737.s007.tif]

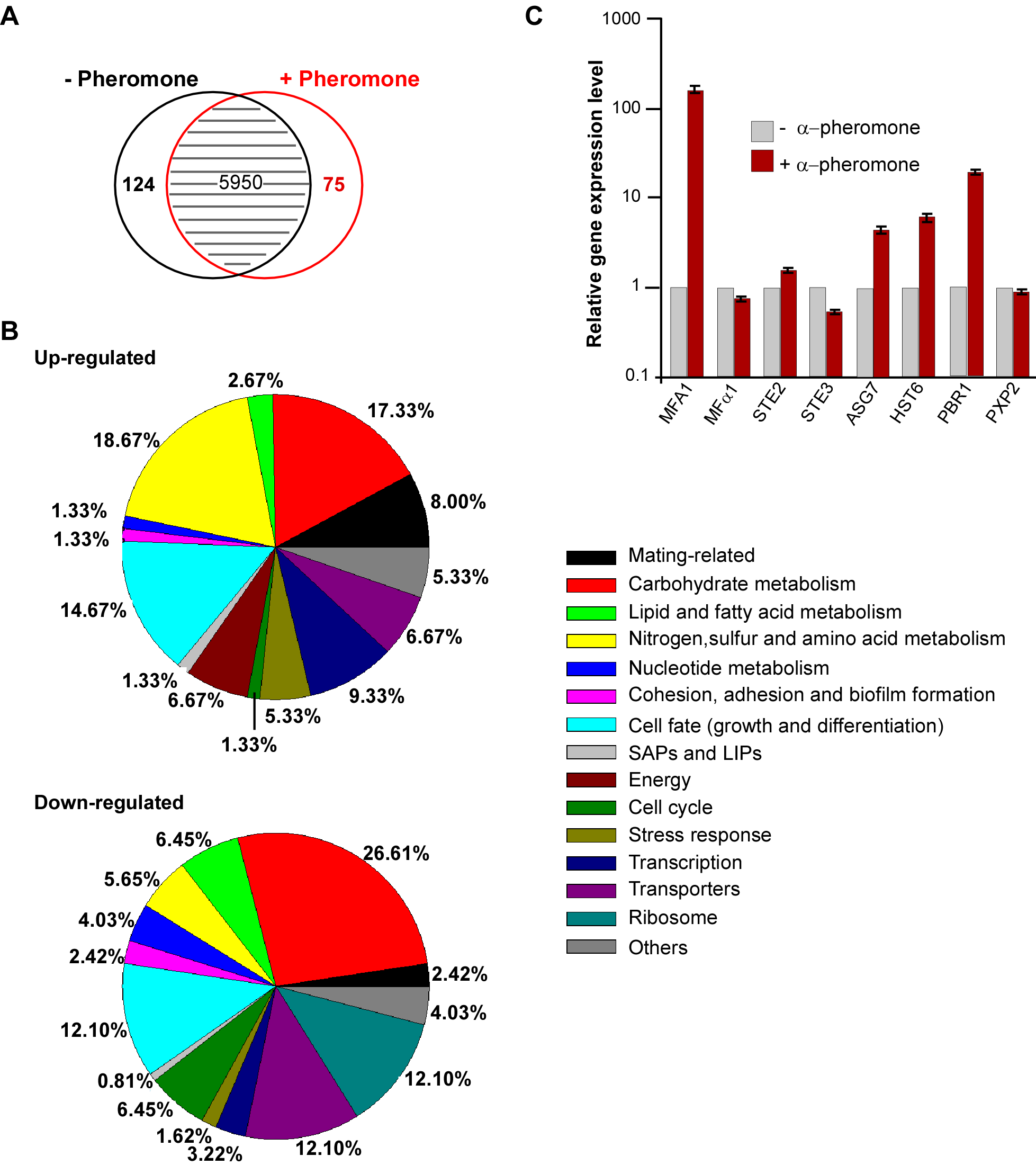

Supplement: Figure S8 — Pheromone-response genes in white cells. (A) Pheromone-up-regulated (75 genes) and down-regulated (124 genes); two-fold threshold cutoff. (B) Functional category of Pheromone-regulated genes. (C) Verification of the relative expression levels of eight pheromone-regulated genes by Q-RT-PCR assays. The wor1Δ/Δ mutant GH1602 was used for RNA-Seq and Q-RT-PCR analysis. The value of the expression level of each gene in pheromone-untreated cells was set as “1”. (TIF) [file pgen.1004737.s008.tif]
