# Supplementary material for: White Cells Facilitate Opposite- and Same-Sex Mating of Opaque Cells in Candida albicans
Source: PLoS Genet. 2014 Oct 16;10(10):e1004737. doi: 10.1371/journal.pgen.1004737 (PMC4199524; doi:10.1371/journal.pgen.1004737)
Supplement: Table S1 — White a cells induce mating projection formation in opaque α cells but mate poorly with opaque α cells. 1×10^6^ white a cells of the WT or wor1Δ/Δ mutant were mixed with 1×10^6^ opaque α cells. The mixtures were spotted onto Lee's glucose medium plates and cultured at 25°C for 48 hours. Mixed cells were replated onto SD medium for prototrophic selection. Mating efficiency and percentages of cells with mating projections for each mixed culture are shown. <, indicates that no progeny colonies (mating efficiency) or no opaque α cells with mating projections were observed. The cross of opaque a cells (GH1012) and opaque α cells (GH1349) served as a positive control. The cross of white a cells (SN152a) and white α cells (SZ306α) served as a negative control. N.A, not analyzed. (DOC) [file pgen.1004737.s009.doc]

**Table S1. White “a” cells induce mating projection formation in opaque “α” cells but mate poorly with opaque “α” cells.**

| **Cross** | **Mating efficiency** | **Perc. of projected opaque “α” cells (%)** |
| --- | --- | --- |
| Wh**a**×Whα (SN152a×SZ306α) | <2.7×10^-8^ | <0.5 |
| Wh**a**×Opα (SZ306a×GH1349α) | <2.6×10^-8^ | >80% |
| Wh**a**×Opα (*wor1*∆/∆×GH1349α) | <3.8×10^-8^ | >80% |
| Op**a**×Opα (GH1012×GH1349α) | 0.2±0.01 | N.A. |

1x10^6^ white **a** cells of the WT or *wor1*∆/∆ (GH1602) mutant were mixed with 1x10^6^ opaque α cells. The mixtures were spotted onto Lee’s glucose medium plates and cultured at 25°C for 48h. Mixed cells were replated onto SD medium for prototrophic growth. Mating efficiency and percentages of “shmooed” cells for each mixed culture are shown. “<”, indicates that no progeny colonies (mating efficiency) or no projected (or shmooed) opaque α cells were observed. The cross of opaque **a** cells (GH1012) and opaque α cells (GH1349) served as a positive control. The cross of white **a** cells (SN152a) and white α cells (SZ306α) served as a negative control. N.A, not analyzed.
