# Supplementary material for: White Cells Facilitate Opposite- and Same-Sex Mating of Opaque Cells in Candida albicans
Source: PLoS Genet. 2014 Oct 16;10(10):e1004737. doi: 10.1371/journal.pgen.1004737 (PMC4199524; doi:10.1371/journal.pgen.1004737)
Supplement: Table S2 — White “a” cells facilitate opposite-sex mating of opaque cells in a mouse skin infection model. Newborn ICR mice (2 to 4 days old) were used for infection. “a-op xα-op” mating cross: 1.2×10^8^ white cells of “helper” strains (∼60%) were mixed with 2.5×10^5^ opaque cells of GH1013h (a/a, his1Δ/Δ) and 8×10^7^ opaque cells of GH1349 (α/α, arg4Δ/Δ). The mixture of opaque “a” cells (GH1013h) and opaque “α” cells (GH1349) served as the control (no “helper” white cells). (DOC) [file pgen.1004737.s010.doc]

**Table S2. White “**a**” cells facilitate opposite-sex mating of opaque cells in a mouse skin infection model.**

| **“Helper” white cells** | **Mating efficiency of**  **a**-op(*his1Δ/Δ*) x α-op(*arg4Δ/Δ*) |
| --- | --- |
| SZ306 (**a**/α, *ura3Δ/Δ wor1Δ/Δ*) | (3.7±6.4)×10^-4^ |
| SZ306a (**a**/Δ *, ura3Δ/Δ wor1Δ/Δ*) | (2.1±0.7)×10^-3^ |
| No “helper” white cells | <1.3 x 10^-3^ |

Newborn ICR mice (2 to 4 days old) were used for skin infection. The mating mixtures were spotted onto the skin on the back of a newborn mouse. “**a**-op xα-op” mating cross: 1.2 x 10^8^ white cells of “helper” strains (~60%) were mixed with 2.5 x 10^5^ opaque cells of GH1013h (**a**/**a**, *his1*∆/∆) and 8 x 10^7^ opaque cells of GH1349 (α/α, *arg4*Δ/Δ). The mixture of opaque “**a**” cells (GH1013h) and opaque “α” cells (GH1349) served as the control (no “helper” white cells). “<”, indicates that “mating efficiency” is less than a certain number or no progeny colonies were observed on selectable plates.
