# Supplementary material for: White Cells Facilitate Opposite- and Same-Sex Mating of Opaque Cells in Candida albicans
Source: PLoS Genet. 2014 Oct 16;10(10):e1004737. doi: 10.1371/journal.pgen.1004737 (PMC4199524; doi:10.1371/journal.pgen.1004737)
Supplement: Table S4 — Strains used in this study. (DOC) [file pgen.1004737.s012.doc]

**Table S4. Strains used in this study**

| **Strain name** | **Parent strain** | **Original background** | **Genotype** | **Reference** |
| --- | --- | --- | --- | --- |
| GH1349 | WUM5A | WO-1 | *MTLα/α ura3::FRT/ura3::FRT arg4::dpl200/ arg4::dpl200-URA3-dpl200* | [1] |
| SZ306 |  | SZ306 | Clinical isolate, *MTL***a**/α | [2] |
| SZ306a | SZ306 | SZ306 | As SZ306, but *MTL***a**/*mtl*α::*FRT-SAT1-FRT* | [2] |
| GH1600 | SZ306a | SZ306 | As SZ306a, but *MFA1/MFA1::MFA1p-GFP* | This study |
| SZ306u | SZ306 | SZ306 | As SZ306, but *ura3::FRT/ura3::FRT* | [2] |
| SZ306u-a | SZ306u | SZ306 | As SZ306u, but *MTL***a**/*mtl*α::*FRT-SAT1-FRT* | This study |
| SZ306u-α | SZ306u | SZ306 | As SZ306u, but *mtl****a****::FRT-SAT1-FRT/MTL*α | This study |
| SZ306w | SZ306u | SZ306 | As SZ306u, but *wor1::URA3/ wor1::FRT-SAT1-FRT* | [2] |
| SZ306w-a | SZ306w | SZ306 | As SZ306w, but *MTL***a***/mtl*α*::FRT-SAT1-FRT* | This study |
| GH1601 (*wor1*∆/∆ **a**/α) | SZ306u | SZ306 | As SZ306u, but *wor1::FRT-SAT1-FRT/ wor1::FRT-SAT1-FRT* | [2] |
| GH1602  (*wor1*∆/∆ **a***/*∆) | GH1601 | SZ306 | As GH1601, but *MTL***a**/*mtl*α::*FRT-SAT1-FRT* | This study |
| GH1603 | GH1602 | SZ306 | As GH1602, but *MFA1/MFA1::MFA1p-GFP* | This study |
| CAI4 | SC5314 | SC5314 | As SC5314, but *ura3*::imm434/ *ura3*::imm434 *iro1/iro1*::imm434 | [3] |
| GH1012 | CAI4 | SC5314 | As CAI4, but *MTL***a**/**a** | [4] |
| BWP17u | CAI4 | SC5314 | As CAI4, but*his1::hisG/his1::hisG::CaHIS1 arg4::hisG /arg4::hisG::CaARG4* | This study |
| GH1013 | CAI4 | SC5314 | As CAI4, but *MTL***a**/**a** *his1*::hisG*/his1*::hisG *arg4*::hisG */arg4*::hisG | [4] |
| GH1013h | GH1013 | SC5314 | As GH1013, but *ura3::imm434/ ura3::imm434::CaURA3 arg4::hisG /arg4::hisG::CaARG4* | This study |
| GH1248  (CAH3) | BWP17 | SC5314 | As GH1013, but *wor1::ARG4/wor1::HIS1* | [5] |
| GH1604  *(wor1*∆/∆ *mfa1*∆/∆) | GH1248 | SC5314 | As GH1248, but *mfa1*::*URA3/mfa1*:: *FRT-SAT1-FRT* | This study |
| SN152 | CAI4 | SC5314 | *As CAI4,* but *URA3/ura3*::imm434 *iro1::IRO1/iro1*::imm434 *his*1::hisG/*his1*::hisG *leu2/leu2 arg4/arg4* | [6] |
| GH1605 | SN152 | SC5314 | As SN152, but *ura3::imm434/ura3::imm434* | This study |
| SN152a | SN152 | SC5314 | As SN152, but *MTL***a**/*mtlα::FRT-SAT1-FRT* | This study |
| GH1606 | SN152a | SC5314 | As SN152a, but *ura3::imm434/ura3::imm434* | This study |
| GH1607 | GH1605 | SC5314 | As GH1605, but *wor1::HIS1/wor1::LEU2* | This study |
| GH1608 | GH1607 | SC5314 | As GH1607, but *MTL***a**/*mtl*α::*FRT-SAT1-FRT* | This study |
| SN152α | SN152 | SC5314 | *As SN152, but mtl****a****::FRT-SAT-FRT1/MTL*α | This study |
| P37005 |  | P37005 | Clinical isolate, *MTL***a***/***a** | [7] |
| L26 |  | L26 | Clinical isolate, *MTL***a***/***a** | [7] |
| MMY288 | CHY420 | SC5314 | *MTL***a***/mtlα1::HisG mtlα2::HisG ura3Δ::imm434/ura3Δ::imm434* | [8] |
| *wor2*∆/∆  (MMY627) | MMY288 | SC5314 | As MMY288, but *wor2::dpl200/wor2::URA3-dpl200* | [8] |
| GH1609  (*mfa1*∆/∆) | GH1013 | SC5314 | As GH1013, but *mfa1::ARG4/mfa1::HIS1* | This study |
| JKC18 | CAI4 | SC5314 | As CAI4, but *cph1::hisG/ cph1::hisG* | [9] |
| GH1610  (*cph1*∆/∆ **a**/∆) | JKC18 | SC5314 | As JKC18, but *MTL***a***/mtl*α*::FRT-SAT1-FRT* | This study |
| GH1611  (*cph1*∆/∆ ∆/α) | JKC18 | SC5314 | As JKC18, but *mtl****a****::FRT-SAT1-FRT/MTL*α | This study |
| JKC131 | CAI4 | SC5314 | As CAI4, but *hst7::hisG/hst7::hisG-URA3-hisG* | [10] |
| GH1613  (*hst7*∆/∆ **a**/∆) | JKC131 | SC5314 | As JKC131, but *MTL***a**/*mtlα::FRT* | This study |
| *ste11*∆/∆ **a**/α | SC5314 | SC5314 | As SC5314, *but ste11::FRT/ ste11::FRT* | [11] |
| GH1614  (*ste11*∆/∆ **a**/∆) | *ste11*∆/∆ **a**/α | SC5314 | As *ste11*∆/∆ **a**/α, but *MTL***a***/mtl*α*::FRT* | This study |
| *ste2*∆/∆ **a**/α | SC5314 | SC5314 | As SC5314, but *ste2::FRT/ ste2::FRT* | [11] |
| GH1615  (*ste2*∆/∆ **a**/∆) | *ste2*∆/∆ **a**/α | SC5314 | As *ste2*∆/∆ a/α, but *MTL***a***/mtl*α*::FRT* | This study |
| GH1616  (*ste3*∆/∆ α/α*)* | WUM5A | WO-1 | As WUM5A, but *ste3::URA3/ste3::FRT-SAT1-FRT* | This study |
| WUM5A | WO-1 | WO-1 | *MTL*α/α *ura3-1::FRT/ura3-2::FRT* | [12] |
| GH1617  (*mfα1*∆/∆ α/α) | WUM5A | WO-1 | As WUM5A, but *mfα1::URA3/mfα1::FRT* | This study |
| GH1247 (JYC13) |  | SC5314 | As CAI4, but *MTL*α/α, *ura3::1imm434/ura3::1imm434 cek1::hisG/cek1::hisG cek2::hisG/cek2::hisG* | [13] |
| GH1618  (*wor1*∆/∆ ∆/α) | GH1601 | SZ306 | As GH1601, but *mtl***a**::*FRT-SAT1-FRT*/*MTL*α | This study |
| GH1619 | GH1618 | SZ306 | As GH1618, but *MFα1/MFα1::MFα1p-GFP* | This study |
| GH1620 | SN152α | SC5314 | AsSN152α, but *MFα1/MFα1::MFα1p-GFP* | This study |

**References**

1. Du H, Guan G, Xie J, Cottier F, Sun Y, et al. (2012) The transcription factor Flo8 mediates CO2 sensing in the human fungal pathogen Candida albicans. Mol Biol Cell 23: 2692-2701.

2. Xie J, Tao L, Nobile CJ, Tong Y, Guan G, et al. (2013) White-opaque switching in natural MTLa/alpha isolates of Candida albicans: evolutionary implications for roles in host adaptation, pathogenesis, and sex. PLoS Biol 11: e1001525.

3. Fonzi WA, Irwin MY (1993) Isogenic strain construction and gene mapping in Candida albicans. Genetics 134: 717-728.

4. Huang G, Srikantha T, Sahni N, Yi S, Soll DR (2009) CO(2) regulates white-to-opaque switching in Candida albicans. Curr Biol 19: 330-334.

5. Huang G, Wang H, Chou S, Nie X, Chen J, et al. (2006) Bistable expression of WOR1, a master regulator of white-opaque switching in Candida albicans. Proc Natl Acad Sci U S A 103: 12813-12818.

6. Noble SM, Johnson AD (2005) Strains and strategies for large-scale gene deletion studies of the diploid human fungal pathogen Candida albicans. Eukaryot Cell 4: 298-309.

7. Pujol C, Pfaller M, Soll DR (2002) Ca3 fingerprinting of Candida albicans bloodstream isolates from the United States, Canada, South America, and Europe reveals a European clade. J Clin Microbiol 40: 2729-2740.

8. Zordan RE, Miller MG, Galgoczy DJ, Tuch BB, Johnson AD (2007) Interlocking transcriptional feedback loops control white-opaque switching in Candida albicans. PLoS Biol 5: e256.

9. Liu H, Kohler J, Fink GR (1994) Suppression of hyphal formation in Candida albicans by mutation of a STE12 homolog. Science 266: 1723-1726.

10. Kohler JR, Fink GR (1996) Candida albicans strains heterozygous and homozygous for mutations in mitogen-activated protein kinase signaling components have defects in hyphal development. Proc Natl Acad Sci U S A 93: 13223-13228.

11. Yi S, Sahni N, Daniels KJ, Lu KL, Srikantha T, et al. (2011) Alternative mating type configurations (a/alpha versus a/a or alpha/alpha) of Candida albicans result in alternative biofilms regulated by different pathways. PLoS Biol 9: e1001117.

12. Strauss A, Michel S, Morschhauser J (2001) Analysis of phase-specific gene expression at the single-cell level in the white-opaque switching system of Candida albicans. J Bacteriol 183: 3761-3769.

13. Chen J, Lane S, Liu H (2002) A conserved mitogen-activated protein kinase pathway is required for mating in Candida albicans. Mol Microbiol 46: 1335-1344.
