# Supplementary material for: White Cells Facilitate Opposite- and Same-Sex Mating of Opaque Cells in Candida albicans
Source: PLoS Genet. 2014 Oct 16;10(10):e1004737. doi: 10.1371/journal.pgen.1004737 (PMC4199524; doi:10.1371/journal.pgen.1004737)
Supplement: Table S5 — Primers used in this study. (DOCX) [file pgen.1004737.s013.docx]

**Table S5. Primers used in this study**

| **Name** | **Sequence (5’ to 3’)** | **Purpose** |
| --- | --- | --- |
| MFA1pGFP-F | TCAAACAAATAACTCTCAATTCTTATAAACTATATTAATTCAATCAACAAATTCAAATAAAAAATGAGTAAGGGAGAAGAACT | MFA1p-GFP reporter |
| MFA1pGFP- R | TGCCATTGATATTGCATTTATATCCTATTGTTGAACAGTGACAGCACCTGTGGCGGTGGCTAGACCACCTTTGATTGTAAATAG |  |
| MFA1pGFP-DF | TTCAGTATGCTTTGGTGTAG | GFP reporter checking |
| GFP-DR | AGCATTGAAGACCATACGCG |  |
| Marker-F | CCGCTGCTAGGCGCGCCGTGACCAGTGTGATGGATATCTGC | Amplification for *ARG4, HIS1, LEU2, URA3* fragments used in fusion PCR |
| Marker-R | GCAGGGATGCGGCCGCTGACAGCTCGGATCCACTAGTAACG |  |
| MFA1-L Fwd | TTAGCTGCATTGTGTCTACC | Fusion PCR for *MFA1* KO |
| MFA1-L rev | CACGGCGCGCCTAGCAGCGGTATGAACTGCAAGAGGTTTG |  |
| MFA1-R Fwd | GTCAGCGGCCGCATCCCTGCTGTCACTGTTCAACAATAGG |  |
| MFA1-R rev | AGTAAACCTGGATTAGAAGC |  |
| MFA1-CHF | TTCAGTATGCTTTGGTGTAG | *MFA1* KO confirmation  (first copy) |
| MFA1-CHR | AATCCTCATTCTGGTTGGTC |  |
| MFA1-orf-F ( MFA1-CHF) | TTCAGTATGCTTTGGTGTAG | *MFA1* KO confirmation  (second copy) |
| MFA1-orf-R | AACAGAACAAGTGGAACAGC |  |
| MFA1-5DR | TCAAACAAATAACTCTCAATTCTTATAAACTATATTAATTCAATCAACAAATTCAAATAAAAATACCGGGCCCCCCCTCGAGGAAGTT | Construction of *wor1*Δ/Δ/*mfa1*Δ/Δ double mutant |
| MFA1-3DR | TGCCATTGATATTGCATTTATATCCTATTGTTGAACAGTGACAGCACCTGTGGCGGTGGCTAGCCGCTCTAGAACTAGTGGATC |  |
| MFα1pGFP-F | AACAATCACCAACAAACTACTAATCACTCTATAACATCAACTAATTAAATCAACAAAAATAACAATGAGTAAGGGAGAAGAACT | MFα1p-GFP reporter |
| MFα1pGFP- R | TACCAATTCATCTAAACAAACATAAAAGTATGATTTCAGTATGCTTTCCATCTTCTTTACTTACGACCACCTTTGATTGTAAATAG |  |
| MFα1-L Fwd | TTCATTTATGCACGTCAAGG | Fusion PCR for  *MFα1* KO |
| MFα1-L rev | CACGGCGCGCCTAGCAGCGGTACTATCTATCCAGTGTATGG |  |
| MFα1-R Fwd | GTCAGCGGCCGCATCCCTGCAGAAGATGGAAAGCATACTG |  |
| MFα1-R rev | AAGATAGGCAAATGCAGAAG |  |
| MFα1-5DR | ACAATCACCAACAAACTACTAATCACTCTATAACATCAACTAATTAAATCAACAAAAATAACATACCGGGCCCCCCCTCGAGGAAGTT | Deletion of the second copy of *MFα1* |
| MFα1-3DR | ACCAATTCATCTAAACAAACATAAAAGTATGATTTCAGTATGCTTTCCATCTTCTTTACTTACGCCGCTCTAGAACTAGTGGATC |  |
| MFα1-CHF | TACATTGTTCTGCAAGTGAC | *MFα1* KO confirmation  (first copy) |
| MFα1-CHR | AGTGAATCACCAGCTTATGG |  |
| MFα1-orf-F | TGCCACTATTGTTGCTGCTG | *MFα1* KO confirmation  (second copy) |
| MFα1-orf-R | AGTCTAAAACCGGCTTCAGC |  |
| STE3-L Fwd | TCTGTCGTCGCTTAGTTAAAC | Fusion PCR for *STE3* KO |
| STE3-L rev | CACGGCGCGCCTAGCAGCGGATCTCCTCGATGTAGTGAATG |  |
| STE3-R Fwd | GTCAGCGGCCGCATCCCTGCTGATCCATCGTATCCTGTTAC |  |
| STE3-R rev | TACGGAACAAGTCAACCAAG |  |
| STE3-5DR | TTTTTAATACCTTAGCATACATAGAGAACTTTATTTTGGCTTCTTAATAATATTTAAAGCAAATACCGGGCCCCCCCTCGAGGAAGTT | Deletion of the second copy of *STE3* |
| STE3-3DR | ATTAGACTTGTTTTTTTTTTATTTATTATATTTTTCATCAACAAGTAACAGGATACGATGGAGCCGCTCTAGAACTAGTGGATC |  |
| STE3-CHF | ATTCTCAGGGGTTGTTTCATC | *STE3* KO confirmation  (first copy) |
| STE3-CHR | ACATCGTTCTGCCAGATATTC |  |
| STE3-orf-F | TTTTGATGAAGCTTGGGATGG | *STE3* KO confirmation  (second copy) |
| STE3-orf-R | AGTGACAAAGGAACCATAGC |  |
| WOR1-L Fwd | AAGAGAAAGAAAGAGAGAGAGG | Fusion PCR for  *WOR1* KO |
| WOR1-L rev | CACGGCGCGCCTAGCAGCGGGATGTTTGAGATGTCAGTGTAC |  |
| WOR1-R Fwd | GTCAGCGGCCGCATCCCTGCATATATGTGGGTCTGTGTGTG |  |
| WOR1-R rev | TCCCTTCATGAATAGTTTCC |  |
| WOR1-CHF | TTGCAGCAACAGATTTCCAC | *WOR1* KO confirmation  (forst copy) |
| WOR1-CHR | TGCATCATAAGGTGAATTCG |  |
| WOR1-orf-F | ATGGTTTCAGCTGCTATACTGC | *WOR1* KO confirmation  (second copy) |
| WOR1-orf-R | ATGATGATTCTGTTTGAGGTGG |  |
| MFA1-RT-F | ATGGCTGCTCAACAACAATC | Q-RT-PCR of *MFA1* |
| MFA1-RT-R | AACAGAACAAGTGGAACAGC |  |
| MFα1-RT-F | TGACAGTAACCAAGTTGTTG | Q-RT-PCR of *MFα1* |
| MFα1-RT-R | AGCACCAGAGGTAAGAGTAG |  |
| STE2-RT-F | TACTGGTTGGTATGATGGATC | Q-RT-PCR of *STE2* |
| STE2-RT-R | AAGGCAACAACAATCAATCC |  |
| STE3-RT-F | TGTTGGTAAGTTGGATGCTG | Q-RT-PCR of *STE3* |
| STE3-RT-R | TGCATATCTTGATCCTGTCAC |  |
| ASG7-RT-F | TTACGACTCTTTGAAAGCTTG | Q-RT-PCR of *ASG7* |
| ASG7-RT-R | TGCCAATGGTTCACTATTTG |  |
| HST6-RT-F | TGTAGCTCAACAATCAATGG | Q-RT-PCR of *HST6* |
| HST6-RT-R | TGATATACAAGACCACTGGAG |  |
| PBR1-RT-F | TGATTACGTTACTCCAGGTG | Q-RT-PCR of *PBR1* |
| PBR1-RT-R | AATACCATCAGAACCAGCAG |  |
| PXP2-RT-F | TGTTGTTTCATCGAGATCAC | Q-RT-PCR of *PXP2* |
| PXP2-RT-R | AATTGTTCAGGTCTGGTTTG |  |
